# Purifying stem cell‐derived red blood cells: a high‐throughput label‐free downstream processing strategy based on microfluidic spiral inertial separation and membrane filtration

**DOI:** 10.1002/bit.27319

**Published:** 2020-03-15

**Authors:** Ewa Guzniczak, Oliver Otto, Graeme Whyte, Tamir Chandra, Neil A. Robertson, Nik Willoughby, Melanie Jimenez, Helen Bridle

**Affiliations:** ^1^ Department of Biological Chemistry, Biophysics and Bioengineering Edinburgh Campus, School of Engineering and Physical Science Heriot‐Watt University Edinburgh Scotland; ^2^ Centre for Innovation Competence – Humoral Immune Reactions in Cardiovascular Diseases University of Greifswald Greifswald Germany; ^3^ Deutsches Zentrum für Herz‐Kreislaufforschung Partner Site Greifswald Greifswald Germany; ^4^ MRC Human Genetics Unit, MRC Institute of Genetics & Molecular Medicine, The University of Edinburgh Western General Hospital Edinburgh Scotland; ^5^ Biomedical Engineering Division, James Watt School of Engineering University of Glasgow Glasgow Scotland

**Keywords:** deformability, purification, sorting, spiral microchannel, stem cell‐derived red blood cells

## Abstract

Cell‐based therapeutics, such as in vitro manufactured red blood cells (mRBCs), are different to traditional biopharmaceutical products (the final product being the cells themselves as opposed to biological molecules such as proteins) and that presents a challenge of developing new robust and economically feasible manufacturing processes, especially for sample purification. Current purification technologies have limited throughput, rely on expensive fluorescent or magnetic immunolabeling with a significant (up to 70%) cell loss and quality impairment. To address this challenge, previously characterized mechanical properties of umbilical cord blood CD34+ cells undergoing in vitro erythropoiesis were used to develop an mRBC purification strategy. The approach consists of two main stages: (a) a microfluidic separation using inertial focusing for deformability‐based sorting of enucleated cells (mRBC) from nuclei and nucleated cells resulting in 70% purity and (b) membrane filtration to enhance the purity to 99%. Herein, we propose a new route for high‐throughput (processing millions of cells/min and mls of medium/min) purification process for mRBC, leading to high mRBC purity while maintaining cell integrity and no alterations in their global gene expression profile. Further adaption of this separation approach offers a potential route for processing of a wide range of cellular products.

## Graphical abstract

To address the challenge of stem‐cell‐derived red blood cells purification, we propose a label‐free approach to separate cells at high throughput based on their morphological (size) and mechanical (deformability) properties. The process consists of two main steps: (a) a microfluidic separation using inertial focusing in spiral microchannel and (b) membrane filtration.

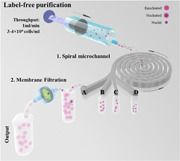

## 1 INTRODUCTION

Stem cell‐derived red blood cells could constitute an attractive pathogen‐free and sustainable alternative for donated blood for rare blood groups and patients requiring regular transfusions (Zeuner et al., 2012). In many cases, such as sickle cell anemia, myelodysplasias and leukemia, multiple blood transfusion is regarded as the only available symptomatic treatment, and that can lead to immunization against the allogeneic red blood cells and transfusion impasses (Douay & Andreu, 2007). The efficient production of manufactured red blood cells (mRBCs) is consequently an ambitious goal for blood services around the world; however, production of a single therapeutic dose (~2 × 10^12^ cells) still remains a significant challenge (Cabrita et al., 2003; Peyrard et al., 2011). Clinical application of mRBC is currently hampered by a lack of technological solutions that would allow production of mRBC at a satisfactory scale and purity in compliance with good manufacture practice (GMP) regulations within the realms of economic feasibility (Bayley et al., 2018; Li, Wu, Fu, & Han, 2013; Migliaccio, Whitsett, Papayannopoulou, & Sadelain, 2012; Rousseau, Giarratana, & Douay, 2014; Shah, Huang, & Cheng, 2014).

To date, mRBC have been produced from several sources of starting material: CD34+ hematopoietic stem cells from peripheral blood (PB) (G. Migliaccio et al., 2002) and umbilical cord blood (CB) (Baek et al., 2008; Fujimi et al., 2008; Neildez‐Nguyen et al., 2002), peripheral blood mononuclear cells (Akker, Satchwell, Pellegrin, Daniels, & Toye, 2010), embryonic (Lu et al., 2008; Ma et al., 2008) and induced pluripotent stem cells (iPSCs; Lapillonne et al., 2010) and recently immortalized adult human erythroid line (Bristol Erythroid Line Adult BEL‐A; Trakarnsanga et al., 2017). In the last 15 years, considerable progress has been achieved in terms of optimizing biological processes underpinning erythroid cell expansion and maturation (Migliaccio, Masselli, Varricchio, & Whitsett, 2012). The selection of starting cell material such as iPSC and BEL‐A cell lines offers a potentially unlimited source for in vitro erythroid differentiation while mitigating blood‐compatibility issues. Moreover, all xenogeneic culture compounds, for example, serum, transferrin, insulin, and growth factors, have been replaced, resolving the associated risks of virus, prions, and immunological complications (Grillberger, Kreil, Nasr, & Reiter, 2009; Migliaccio et al., 2010; Miharada, Hiroyama, Sudo, Nagasawa, & Nakamura, 2006; Timmins, Athanasas, Gunther, Buntine, & Nielsen, 2011). A mini‐transfusion (10^11^ cells) of autologous CB CD34+ derived mRBC (under GMP conditions) was given to a patient in 2011, providing the proof of principle of mRBC feasibility for clinical use (Giarratana et al., 2011; http://www.clinicaltrials.gov; Emelyanenko, 2009).

**Figure 1:**
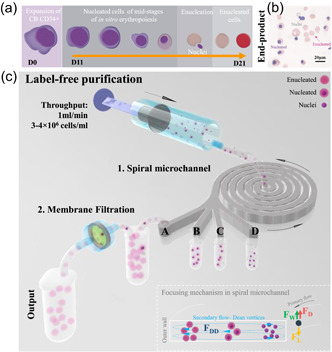
(a) Cord blood (CB) CD34+ cells undergo in vitro differentiation into manufactured red blood cells (mRBCs) over the course of 21 days. (b) As shown in the exemplary cytospin image, the end‐product of the differentiation protocol is a heterogeneous population containing enucleated mRBC, partially differentiated or undifferentiated nucleated cells as well as free‐floating nuclei. The scale bar corresponds to 20 µm. (c) The proposed label‐free sorting strategy for the end‐product consists of two steps: first, the sample is processed in a spiral microchannel with a rectangular cross‐section (170 × 30 um^2^), six loops, one inlet, and four outlets (A, B, C, and D). Inertial focusing within spiral microchannels occurs due to balance of shear gradient lift force (F_L_), wall‐induced lift force (F_W_) as well as Dean drag (F_DD_). Particles of different sizes interact with a different section of the characteristic cross‐sectional velocity profile (Dean vortices). Deformable particles experience an additional deformability‐induced lift force (F_D_). Cells align in the spiral channel at distinct lateral equilibrium positions, that facilitates their capture in one of the four outlets. The majority of the desired enucleated cells is captured in outlet A with some contaminant nucleated cells, which are further removed by membrane filtration [Color figure can be viewed at wileyonlinelibrary.com]

Despite considerable progress in improving the expansion rate and yield of mRBC, enucleation rates remain limited (Table S1). The end‐product of existing differentiation protocols is consequently a heterogeneous mixture of enucleated mRBC, nucleated cells that remain at earlier developmental stages and free‐floating nuclei expelled during the enucleation process (Figure 1b). In 2008, Fujimi et al. (2008) reported a differentiation strategy with CB CD34+ providing an almost complete enucleation (99.4%) (Fujimi et al., 2008). However, this was achieved by coculture with macrophages, making the protocol challenging to scale‐up (Goers, Freemont, & Polizzi, 2014). With lower enucleation rates, the presence of residual nucleated cells and expelled nuclei constitute a potential danger if intended for transfusion into patients (Bouhassira, 2008; Guzniczak et al., 2017). Undifferentiated nucleated cells can give rise to teratomas (benign tumors of differentiating cells) and teratocarcinomas (malignant metastatic tumors composed of highly proliferative cells; McGowan, Campbell, & Mountford, 2018), thus they have to be removed from the sample and require adequate purification approaches. In addition, presence of free‐floating nuclei in large quantities may prove particularly problematic in large‐scale culture systems by fouling surfaces and entangling the desired cell product in DNA (Timmins & Nielsen, 2011)

Traditionally, cell purification is performed using fluorescent or magnetic activated cell sorting (FACS or MACS) (Schriebl, Lim, Choo, Tscheliessnig, & Jungbauer, 2010). FACS and MACS both generate highly defined, purified (>95%) cell populations with a low number of unwanted cells in the final product; however, the requirement for cell‐specific ligands hinders adaptation of these methods to industrial‐scale processing due to the high cost of antibodies. In addition, immunolabeling is a laborious multi‐step process consisting of numerous centrifugation, washing, and incubation steps often resulting in a significant (reported up to 70%) cell loss (Schriebl et al., 2010) and post‐isolation cell quality impairment (Lee et al., 2018). Currently, only a limited number of fluorophore‐conjugated antibody reagents are suitable for clinical processing (McIntyre, Flyg, & Fong, 2010) and the adverse effects of introducing these probes into patients are unknown, but it is generally recognized that they could potentially trigger immune and toxic responses (Willoughby et al., 2016). Various alternatives to FACS and MACS for cellular therapies, such as mRBC production, have been proposed and were recently reviewed (Masri, Hoeve, Sousa, & Willoughby, 2017). Recent work on deterministic lateral displacement (Campos‐Gonzalez et al., 2018) and inertial vortexes (Pritchard et al., 2019) have demonstrated application to CAR‐T cell processing and inertial focussing has been used to isolate, enrich, and purify stem cells (Hur, Brinckerhoff, Walthers, Dunn, & Di Carlo, 2012; Lee et al., 2018; Song et al., 2017), to obtain desired subpopulation (Lee et al., 2011, 2014; Poon et al., 2015), to isolate single cells from clusters (Nathamgari et al., 2015), for nonviable cell removal (Kwon, Yao, Hamel, & Han, 2018) as well as microcarrier scaffold removal (Moloudi et al., 2018).

To address the challenge of mRBC purification, we propose a label‐free approach to separate cells at high throughput based on their morphological (size) and mechanical (deformability) properties. As presented in Figure 1c, the process consists of two main steps: (a) a microfluidic separation using inertial focusing in spiral microchannel and (b) membrane filtration. Due to its simplicity in operation, low manufacturing cost and proven scalability by parallelization (allowing processing millions of cells per minute) inertial focusing in spiral channels has been recognized as an attractive approach for high‐throughput cell sorting (Gou, Jia, Wang, & Sun, 2018) for a wide range of applications (for a comprehensive review, see Gou et al., 2018). Traditionally, spiral microchannels have been used for sorting cells based on size differences. Cells traveling within the channel experience inertial lift force (combination of shear gradient lift force [F_L_], wall‐induced lift force [F_W_], and Dean drag [F_DD_]), and if cells travel a long enough distance, these forces balance, focusing cells at distinct lateral equilibrium positions (measured as a distance from the outer wall) depending on their size (Bhagat, Kuntaegowdanahalli, & Papautsky, 2008; Gou et al., 2018). As shown in Figure 1c, smaller nuclei are positioned closer to the inner wall while larger cells are observed closer to the channel centreline. As we previously reported (Guzniczak et al., 2020), there is a distinct hydrodynamic behavior of cells of the same size but different deformability at sufficiently elevated flowrate. Stiff cells remain focused close to the inner wall, while their softer counterparts experience additional drag (as a consequence of the additional deformability‐induced lift force [F_D_]; Hur, Henderson‐MacLennan, McCabe, & Di Carlo, 2011) and they travel across the channel to be equilibrated near the outer wall. Particles of the same size but different deformability assemble at distinct equilibrium positions within the channel cross‐section, hence cell deformability can also be used as a sorting parameter.

In this study, the impact of deformability on the focusing mechanism has been translated into an effective label‐free purification protocol for mRBC derived from cord blood CD34+ cells. This approach offers a viable alternative to FACS and MACS for sorting mRBC at industrial scale in a label‐free manner at high purities and without compromising cell quality, consequently creating a new route to bring mRBC into clinical use.

## 2 MATERIALS AND METHODS

### 2.1 Differentiating CD34+ into red blood cells

The differentiation protocol was performed in accordance with relevant guidelines and regulations and was approved by the Heriot‐Watt Engineering and Physical Sciences Ethics Committee as well as the Heriot‐Watt Engineering and Physical Sciences Biosafety Review. Umbilical cord blood CD34+ cells were purchased from Stem Cell Technologies and differentiated using a modified version of a protocol described previously (Griffiths et al., 2012). Cells were cultured for 21 days in basal growth medium: Iscove's basal medium (cat. BCHRFG0465; VWR), 5% human AB+Serum (cat. H4522; Sigma‐Aldrich), 3 U/ml heparin (cat. H5515; Sigma‐Aldrich), 10 µg/ml insulin (cat. 19278; Sigma‐Aldrich), and 200 µg/ml human holotransferrin (cat. 616397‐500; VWR) supplemented as outlined in Table 1. At each passaging occasion (Days 8, 11, 14, and 18), cells were harvested by centrifugation at 200*g* for 5 min and resuspended in fresh medium supplemented with appropriate compounds. All cell culture manipulations were carried under aseptic conditions in a cabinet with laminar air flow.

**Table 1:** Changing cell culture medium composition for the 21 days CB CD34+ differentiation protocol

Day	Medium composition
0–8	60 ng/ml recombinant human stem cell factor (SCF) (cat. 300‐07; PeproTech)
	5 ng/ml recombinant human IL‐3 (cat. 200‐03; PeproTech)
	3 U/ml erythropoietin (EPO) (clinical grade material; Roche)
	1 µM hydrocortisone (cat. H0888; Sigma‐Aldrich)
8–14	10 ng/ml SCF
	3 U/ml erythropoietin
	1 µM hydrocortisone
	300 µg/ml holotransferrin
14–21	3 U/ml erythropoietin
	300 µg/ml holotransferrin

### 2.2 Cell characterization

#### 2.2.1 Flow cytometry

Each population is characterized by a combination of molecular markers such as presence/absence of DNA (DNA+/DNA−) and expression/lack of expression of glycophorin A (CD235a+/CD235a−). Enucleated cells are DNA− and CD235a+, nucleated cells are DNA+ and CD235a+, free‐floating nuclei are DNA+ and CD235a+, however, they express lower levels of CD235a than nucleated cells. Each 100 µl aliquot of cells (~1 × 10^6^ cell/ml) suspended in phosphate‐buffered saline (PBS) without calcium and magnesium (PBS−/−; Gibco) was supplemented with 0.5% bovine serum albumin (Sigma‐Aldrich), 0.625 µl of fluorescein isothiocyanate‐conjugated Mouse Anti‐Human CD235a (cat. 559943; BD) and 0.5 µl of 5 mM DRAQ5™ Fluorescent Probe (cat. 564902; BD). Cells were incubated for 20 min at room temperature in darkness and the excess fluorescent stain was not removed to prevent cell damage. Cells were analyzed on a flow cytometer (BD LSR II; BD) and data processed using FlowJo V10 CL and GraphPad Prism 6.

#### 2.2.2 Real‐time fluorescence and deformability cytometry

**Figure 2:**
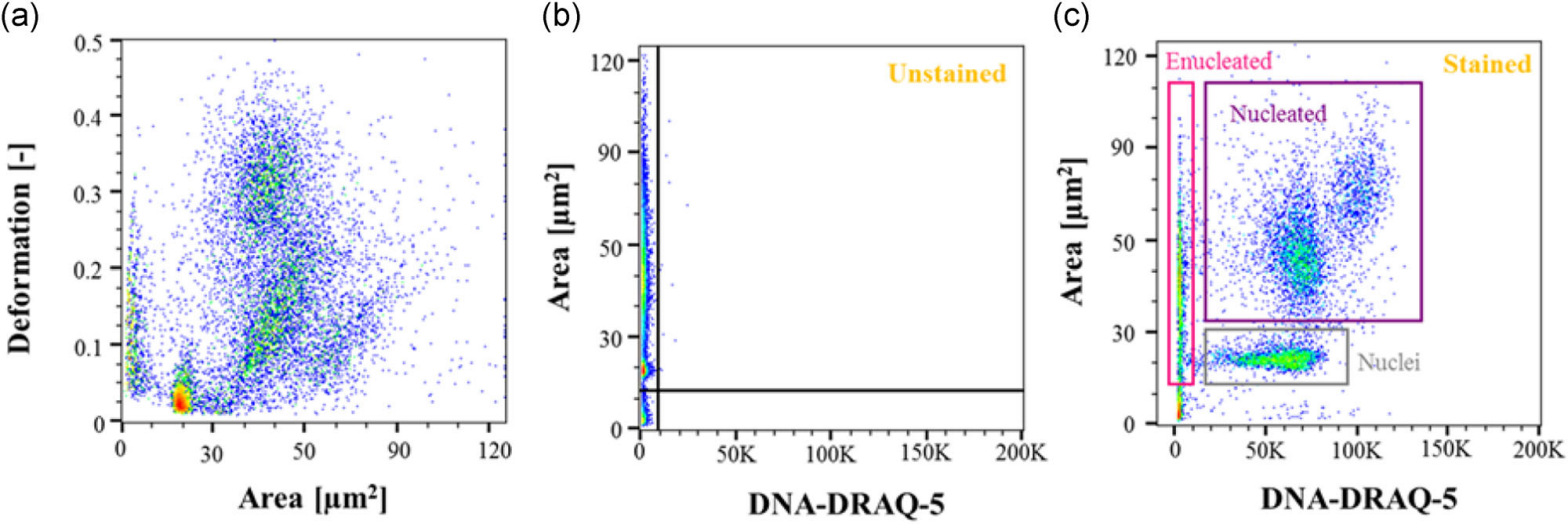
Gating strategy applied to characterize the end product of CB CD34+ in vitro erythropoiesis. The sample collected at the end of the differentiation protocol was stained with a nuclear stain DRAQ5 to check for the presence of a nucleus. Each subpopulation can be characterized by a combination of size and fluorescent signal. Enucleated cells are inherently negative for DNA (DRAQ5‐DNA−), nucleated cells are larger than the free‐floating nuclei and both are DRAQ5‐DNA+. Events between 0 and 15 µm^2^ were assumed to be cell debris and they were excluded from the analysis. (a) Scatter plot of the area (µm^2^) versus deformability (−) for a control unstained sample for more than 20,000 acquired events. (b) Scatter plot of DRAQ5‐DNA versus area (µm^2^) for the unstained sample. The gate splits the scatter plot into DNA‐negative region on the left hand side and DNA‐positive region on the right hand side. (c) Scatter plot for the sample stained with DRAQ5 for the presence of DNA. Gates for each subpopulations are shown as color‐coded rectangles: pink for enucleated cells, purple for nucleated cells and gray for nuclei [Color figure can be viewed at wileyonlinelibrary.com]

Cells' morphological and mechanical properties were assessed using real‐time fluorescence and deformability cytometry (RT‐FDC; Rosendahl et al., 2018) (for detailed description of the technique, see Supporting Information Material). Briefly, CB CD34+ cells were harvested at the end of the differentiation protocol by centrifugation at 200*g* for 5 min and resuspended in a 0.05% methylcellulose solution (CellCarrier; Zellmechanik Dresden, Germany) to reach a final concentration of 1–2 × 10^6^ cells/ml. Due to their fragile nature, cells were stained directly in CellCarrier by adding 5 mM DRAQ5™ Fluorescent Probe (BD) (to obtain a final concentration of 5 µM) per 100 µl buffer volume. Cells were incubated for 2 min, in darkness at room temperature and analyzed immediately after staining. CB CD34+ cells were injected in a 20 × 20 µm cross‐section channel at 0.12 µl/min for real time size and deformability measurement. The gating strategy for enculated/nucleated cells and nuclei is detailed in Figure 2 with data obtained using the RT‐FDC software ShapeOut 0.8.4 (available at www.zellmechanik.com).

### 2.3 Cell morphology—cytospin

To visualize cells' morphology and structure, cells were transferred onto microscope slides using a cytocentrifuge then fixed and stained using Giemsa‐Wright staining (Rapid Romanowsky Stain Pack, cat. SW167/500; TCS Bioscience). Cells were harvested by centrifugation at 300*g* for 5 min and resuspended at 2 × 10^6^ cells/ml in PBS−/− (Dulbecco's PBS buffer without calcium and magnesium; Gibco). One hundred microliters of cell suspension was transferred into a cytocentrifuge cell funnel and centrifuged at 450 rpm for 4 min in a cytocentrifuge (Cellspin I; Tharmac, Germany) to transfer the cells onto the slide. Slides were then air‐dried for 15 min, fixed, and stained according to the manufacturer's instructions. After staining, slides were air‐dried, then fixed with DePeX mounting medium (cat. 06522; Sigma‐Aldrich). Slides were photographed for further image analysis using either an EOS 60D Canon camera (Canon, UK) mounted on an AXIO Scope.A1 Zeiss microscope (Zeiss, Germany) at ×100 magnification or using a Canon 650d camera (Canon) mounted on a Motic AE31 microscope (Motic, UK) at ×40 magnification. Images were analyzed in either Matlab R2016b using a custom‐made script or using bespoke LabView software, which detected the outline of the cells and nuclei by thresholding. The detected objects were classified into nucleated cells, enucleated cells, and free‐floating nuclei, and the measurements of the morphological features were extracted for further processing.

### 2.4 Separation in spiral channels

#### 2.4.1 Microfluidic system

To sort mRBC from contaminant nucleated cells and free‐floating nuclei a spiral channel with a rectangular cross‐section (30 µm deep and 170 µm wide), six loops, one inlet, and four balanced outlets (A, B, C, and D) were used (Figure 1 and Figure S10). Due to the laminar flow regime, fluid flowing thought the channel is split into four equal portions, flowing with the same volumetric throughput into the corresponding outlets. Microfluidic devices were fabricated by lithography in Poly(methyl methacrylate) (PMMA; Epigem, UK).

#### 2.4.2 Cell processing

The current differentiation protocol involves the use of human serum as a supplement to cell culture media. It provides high concentrations of growth factors, macromolecules, carrier proteins for lipids, trace elements, attachment and spreading factors, nutrients, and hormones (Heger et al., 2018). We however found, similarly to others (Henderson et al., 2010), that microfluidic channels can clog with serum (Henderson et al., 2010) and recommend using a serum‐free buffer for processing. Moreover, the presence of phenol red (pH indicator in basal medium) impairs reads from both flow cytometry and automated cell count, thus cells processed in the basal medium could not be directly sampled for the quality control tests. In this study, we used PBS−/− supplemented with 0.1% biocompatible surfactant Pluronic F‐68 (Guzniczak et al., 2018) (cat. 24040032; Thermo Fisher Scientific) as a processing buffer. Pluronic F‐68 was added to surrogate the serum protective mechanism from mechanical damages (e.g. due to shear stress generated within the spiral microchannel) (Heger et al., 2018; Tharmalingam, Ghebeh, Wuerz, & Butler, 2008; Guzniczak et al., 2018).

Cells suspended in PBS−/− supplemented with 0.1% Pluronic F‐68 at circa 3–4 × 10^6^ cells/ml were injected in a spiral microfluidic channel with a mid‐pressure syringe pump (neMESYS 1000N; Cetoni, Germany) in 10‐ml batches trough 1/16” PTFE tubing of 0.5 mm internal diameter (Thames Restek, UK). Cell concentration is a critical factor influencing focusing within the spiral microchannel. If the concentration is too high, the steric crowding effect occurs, meaning that there is physically not enough space for particles to focus in a tight single stream. To identify if the crowding effect will occur, the parameter α (number of particle diameters per channel length) can be calculated.

(1) $$\alpha =\frac{6\mathrm{WH}V_{F}}{\pi a^{2}},$$

where W (resp. H) the width (resp. height) of the channel cross section, $V_{F}$ is the volume fraction of particles in the solution, a the particle diameter. For α > 1, focusing to a single stream can be challenged by steric interactions between particles (Di Carlo, 2009). Assuming that all the particles in the input sample were of the size of the largest nucleated cells (a~5 µm), at 3–4 × 10^6^ cells/ml, α varies between 0.153 and 0.255 giving an upperbound of α < 1.

As shown in Table 2, cells were examined at flow rates ranged from 200 to 1,000 µl/min (corresponding to channel Reynolds number [Re] between 33 and 168, Dean number [*De*] ranging between 5 and 26).

**Table 2:** Table summarizing experimental conditions (applied flow rates and corresponding velocities, Reynolds number [*Re*], and Dean number [*De*])

Flow rate (ml/min)	Velocity (m/s)	*Re* (‐)	*De*
0.2	0.65	33	5
0.4	1.3	66	10
0.6	1.9	97	15
0.8	2.6	132	21
1	3.3	168	26

The channel Re is a dimensionless parameter, which describes the unperturbed channel flow.

(2) Re=Inertial⁢ forcesViscous⁢ forces=ρUDhμ,

where ρ is the medium density, Uis the medium velocity, μ is the dynamic viscosity, and $D_{h}$ is the hydraulic diameter, defined as

(3) $$D_{h}=\frac{2\times H\times W}{H+W},$$

where H is the channel height and Wis the channel width.

*De* is used to quantify the secondary flow within spiral microchannel, and it is defined as

(4) De=ReDhR,

where *R* is the radius of the curvature.

The focusing behavior of cells was assessed in terms of lateral equilibrium position, measured as a distance from the cell center to the outer wall of the spiral channel in region of interest as shown in Figure S10B. Images of cells inside the spiral channel were recorded at ×10 magnification with a 4.9 mm free working distance (421251‐9911‐000 LD A‐Plan 10× Ph1; Zeiss) using high‐speed camera (CCD ProgRes®; Jenoptik, Germany) mounted on a microscope (Zeiss Axio Observer 3; Zeiss). Images were recorded at 130 frames per second and analyzed using a bespoke MatLab script. To enhance the purification efficiency, cells collected in outlet A of the spiral channel were passed through a 3 µm polycarbonate Isopore™ filter membrane (Merc, UK). Cell suspension was loaded into a 5 ml plastic syringe and pumped through the filter membrane at 2 ml/min using a syringe pump (neMESYS; Cetoni). Cell suspensions were passed through the filter membranes fitted onto syringe adapter and the filtrate was collected in 10‐ml plastic tube. The sorting performance was assessed using the following three parameters:

(5) $$\text{Separation}\,\;\text{efficiency}{\left[C_{\text{type}}\right]}_{{\text{outlet}}_{i}}=\frac{{\left[C_{\text{type}}\right]}_{{\text{outlet}}_{i}}}{\sum_{i=1}^{4}{\left[C_{\text{type}}\right]}_{{\text{outlet}}_{i}}}$$

of each cell type in each outlet, where $\left[C_{\text{type}}\right]$ is the concentration of given cell type cells in a given outlet *i* (*i* =A, B, C, or D).

(6) $${\text{Purity}[C_{\text{type}}]}_{{\text{outlet}}_{i}}=\frac{{\left[C_{\text{type}}\right]}_{{\text{outlet}}_{i}}}{{\left[C_{\text{all}}\right]}_{{\text{outlet}}_{i}}}\times 100\%$$

indicating a fraction of each subset in a sample collected after processing, where $\left[C_{\text{all}}\right]$ is the concentration of all cell types found in the sample and

(7) $$\text{Enrichment}\,\;\text{ratio}=\frac{{\left[C_{\text{type}}\right]}_{{\text{outlet}}_{i}}}{{\left[C_{\text{type}}\right]}_{\text{inlet}}}.$$

Cell separation efficiency was quantified by flow cytometry (BD LSR II; BD) to compare the fraction of each cell population (characterized by unique fluorescent properties) in samples collected at each outlet and after filtration. In addition, cell yield was assessed by counting the number of cells at each outlet and after filtration using MoxiZ automated cell counter (Orflo). Further data analysis was performed using GraphPad Prism 6 and FlowJo V10 CL.

In terms of actual recovery, determined by total number of cells collected versus total number of cells injected into the system, there is a slight variation (~5%) since the sample is subjected to dilution and sedimentation. Before the procedure, device is primed with running buffer without cells, the dead volume was assessed as 1.5 m, which should be discarded, since it takes around 1 min for the system to stabilize, and we collect cell suspension after this time. Since the processing time for one batch is 10 min, the first portion of collected suspension is slightly more concentrated than the very last one due to sedimentation.

#### 2.4.3 Viability—trypan blue exclusion assay

Control cells were not passed through the device but they were incubated on a bench outside of an incubator for the time of the treated (spiral) sample processing in the spiral microchannels. Both control and treated cells were recultured under normal conditions for 1 hr, before the trypan blue viability assays: 100 µl of cell suspension (control/treated cells) was mixed with an equal part of 0.4% trypan blue dye (Gibco, UK), incubated for less than 3 min at room temperature, loaded onto a glass haemocytometer and counted using a light microscope (AXIO Scope.A1; Zeiss).

#### 2.4.4 Global gene expression

To assess whether cell processing within the spiral microchannel results in global gene expression alterations, CB CD34+ cells undergoing in vitro erythropoiesis collected at Day 14 of the differentiation protocol were used. Both control (not passed through the device but they were incubated on a bench outside of an incubator for the time of the treated sample processing in the spiral microchannels) and treated cells after processing were recultured under normal conditions for 24 hr and then harvested by centrifugation at 200*g* for 5 min. The cell pellets were flash‐frozen by immersing in liquid nitrogen batch and stored at −80°C until ready for RNA extraction (performed using MagMAX™ mirVana™ Total RNA Isolation Kit; Thermo Fisher Scientific, as indicated by the instruction manual). The impact of filtration was not studied.

The global gene expression measurements by poly‐A selection were performed by the Edinburgh Clinical Research Facility (https://www.edinburghcrf.ed.ac.uk/Genetics) and run according to their internal standard operating procedures. The bioinformatic analysis comprised of trimming N bases and the filtering of poor‐quality reads (Phred‐score ≤ 30) with trim‐galore before aligning with HISAT2 to the GRCh38 human genome (Ensembl version 94). Post alignment, reads were sorted with SAMtools before quantifying explicit normalized expression with the Cufflinks suite. Differential expression analysis was performed using CuffDiff where significance was determined at a false discovery rate ≤ 0.05 cut‐off.

## 3 RESULTS

### 3.1 Manufactured red blood cell purification

#### 3.1.1 Sample characterization

**Figure 3:**
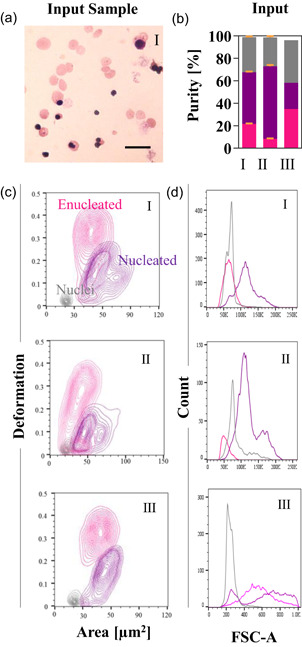
The end product of CB CD34+ derived from donor I, II, and III‐ in vitro differentiation into red blood cells. (a) Exemplary image of the end product (input sample) for cells derived from donor I. Scale bars correspond to 20 µm. (b) Size and deformability. (c) Equal probability contour plots (the same number of cells fall between each pair of contour lines) of deformation vs cell size (expressed as projected cell area in µm^2^) for enucleated (pink) and nucleated (purple) cells and nuclei (gray). (d) Histograms of FSC‐A parameter reflecting relative sizes of enucleated and nucleated cells as well as the free‐floating nuclei, measured by flow cytometry. The number of events on each diagram is around 10,000—split accordingly between each subpopulation [Color figure can be viewed at wileyonlinelibrary.com]

The first step to develop the label‐free strategy for purifying mRBC from the contaminant nucleated cells and free‐floating nuclei was to identify if there was a unique set of label‐free markers, such as cell size and deformability that would allow characterization of each of the subsets within the final product. These findings are detailed for donor III in Guzniczak et al. (2017) and for donors I and II in Table S3 (for the convenience of the reader, also, data on donor III are included in STable 3). The study was further conducted here for cells from three different donors (see Figure 3 for exemplary data of one replica from each donor).

As presented in Figure 3 and Table S3, the relative size measured by flow cytometry as FSC‐A parameter shows that nucleated cells always remain the largest in the samples (except for samples derived from donor III, where they significantly overlap in size with enucleated cells) and that was true across cells sourced from the three different donors, nuclei always remain the smallest and the most rigid, while the enucleated cells are the most deformable. Across the three researched donors, there were discrepancies in the relative size of enucleated cells and nuclei. Enucleated cells derived from donor I substantially overlapped with sizes of nuclei (area under the curve [AUC] = 0.68, Table S3) and the little shift was toward the larger side of the size spectrum. For donor II, enucleated cells were the smallest within the sample, while the subpopulation of enucleated cells derived from donor III was larger than the subpopulation of the expelled nuclei (AUC = 0.95) with a substantial overlap in terms of size with nucleated cells (AUC = 0.56). For all the three donors, a separation based solely on size would consequently lead to a relatively high likelihood of contamination by nucleated cells or nuclei—the heterogeneity in cell size from one donor to another could also be a challenge for channel design and large scale processing. However, all the three donors lead to mRBC (enucleated cells) that could be purified if sorting was based on deformability. There were discrepancies between microscopy measurements of size and those determined by FSC‐A, most probably the consequence of the nature of sample preparation for each technique. In contrast to cytospin, flow cytometry allows size measurement for live cells in suspension. FSC intensity produces a voltage which is proportional to the cell diameter, however, cell size is not reported in physical units. In the cytospin technique, deformable enucleated cells spread on the slide more than rigid nuclei, consequently appearing larger than they really are. Thus, if the physical value plays an important role (e.g., for fine‐tuning sorting device dimensions), a supplementary approach to flow cytometry is required.

#### 3.1.2 Process optimization

**Figure 4:**
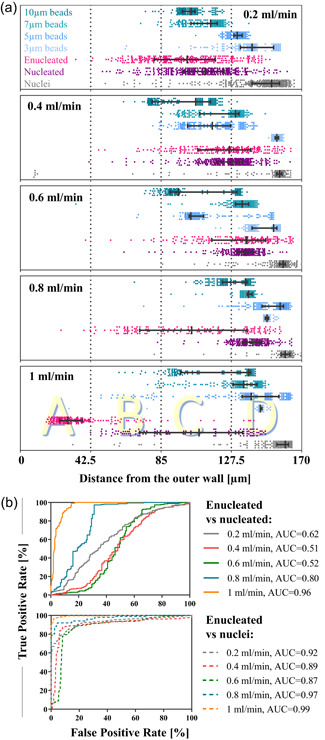
(a) The hydrodynamic behavior of presorted on fluorescent activated cell sorting pure populations of enucleated (pink) and nucleated (purple) cells and free‐floating nuclei (gray) as well as 3, 5, 7, and 10 µm beads (shades of blue) was assessed in a spiral microchannel with a 30 × 170 µm^2^ cross‐section at five different flow rates: 0.2, 0.4, 0.6, 0.8, and 1 ml/min. Lateral equilibrium positions are measured as a distance from the outer wall (µm) at the end of the spiral channel. Data reported as median (represented as the longest vertical line) and the interquartile range (indicated by the short vertical lines) on top of scatter plots, where each dot represents one event. Around 200 events are shown for each subpopulation. Vertical dotted lines indicate four sections of the channel corresponding to four outlets of the channel (0–42.5 µm: outlet A, etc.). (b) Receiver operating characteristic curves were plotted for lateral equilibrium position for enucleated cells versus nucleated cells and enucleated cells versus nuclei for each applied flowrate. The true positive rate is defined as the number of enucleated cells found at a given lateral position and divided by the total number of enucleated cells. The false positive rate is the corresponding number of nucleated cells (resp. nuclei) divided by the total number of nucleated cells (resp. nuclei) for the same cut‐off. To determine which of the applied flow rate ensures the best separation efficiency the area under the curve was calculated [Color figure can be viewed at wileyonlinelibrary.com]

To identify optimal conditions to take advantage of deformability for mRBC purification, the performance of a spiral microchannel with 170 × 30 µm^2^ cross‐section has been tested with FACS presorted pure populations of enucleated and nucleated cells and nuclei from donor III. As mentioned previously, nucleated and enucleated cells sourced from this donor have a significant overlap in their size and require a different strategy for sorting. Each subpopulation (enucleated cells, nucleated cells and nuclei—sorted using FACS) was run separately at gradually increasing flow rates (0.2, 0.4, 0.6, 0.8, and 1 ml/min; Figure 4a). The separation potential of enucleated cells from nucleated and nuclei was estimated for each tested flow rate by generating receiver operating curves curves and calculating the AUC (Figure 4b). At lower flow rates (0.2, 0.4, and 0.6 ml/min), all three subpopulations were pushed toward the inner wall. Enucleated and nucleated cells occupy the same section of the channel (enucleated: 100 ± 24 µm, 123 ± 22 µm, and 131 ± 23 µm; nucleated: 110 ± 20 µm, 125 ± 15 µm, and 134 ± 11 µm [mean ± *SD*] at 0.2, 0.4, and 0.6 ml/min, respectively) with a substantial overlap in the lateral equilibrium position (AUC = 0.62, 0.51, and 0.52, for 0.2, 0.4, and 0.6 ml/min, respectively) closer to the channel centreline. Increasing the flow rate to 1 ml/min triggered the characteristic shift, recently reported and characterized in Guznizcak et al. (2020) of more deformable enucleated cells toward the outer wall to the equilibrium lateral position at 36 ± 21 µm, while less deformable nucleated cells recapitulated the unfocused transition pattern (103 ± 32 µm) observed for enucleated cells at 0.8 ml/min (103 ± 33 µm). Except for the lowest applied flow rate, nuclei always remained focused along the inner wall in a tight stream (151 ± 23 µm at 0.4 ml/min, 156 ± 11 µm at 0.6 ml/min, 158 ± 11 µm at 0.8 ml/min, and 154 ± 18 µm at 1 ml/min). In conclusion, theoretically, operating at 1 ml/min flow rate would allow separation of 96% of nucleated cells (AUC = 0.96) and 99% of nuclei (AUC = 0.99%) from the enucleated cells.

#### 3.1.3 Process performance

**Figure 5:**
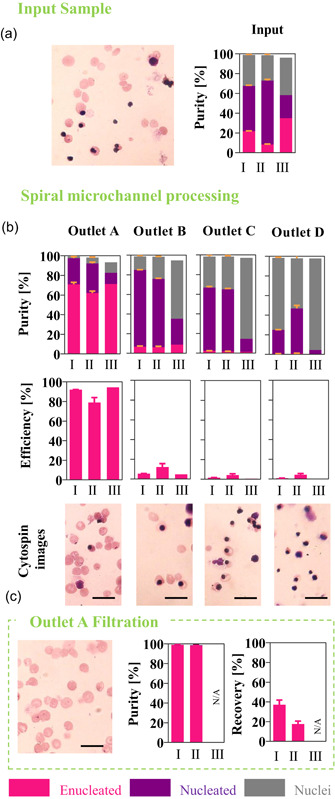
The label‐free purification process of the end‐product of CB CD34+ derived from donors I, II, and III‐ in vitro differentiation into red blood cells, has been designed after (a) input sample characterization in terms of purity. Exemplary image of the end product (input sample) for cells derived from donor I. Scale bars correspond to 20 µm. (d) Characterization of the label‐free purification process for mRBC derived from three donors (indicated as I, II, and III). The separation efficiency of enucleated cells from contaminant nucleated cells and nuclei after processing in a spiral channel with a 30 × 170 µm^2^ cross‐section and four outlets (A ‐closest to the outer wall, B, C, and D) at 1 ml/min flow rate, and enhanced by filtration. (e) The filtration step was performed only on cells collected at the best performing outlet A. Both steps were characterized by calculating purity and efficiency. The process validation was performed with three replicas for donor I and II and one, the bars representing the mean value and error bars the standard error of the mean. Exemplary images for cells derived from donor I, are shown for each step (input sample, postprocessing in spiral microchannel and filtration). Scale bars correspond to 20 µm. mRBC, manufactured red blood cell [Color figure can be viewed at wileyonlinelibrary.com]

Differences in hydrodynamic behavior of enucleated and nucleated cells, as well as nuclei observed at 1 ml/min flow rate, were translated and incorporated into a label‐free purification process for mRBC, derived from three donors (indicated as donor I, II, and III). The heterogeneous end‐product after the differentiation protocol was injected into the spiral microchannel at 1 ml/min at a concentration of around 3 × 10^6^ cells/ml. Figure 5a shows an averaged fraction of each subset in the input sample derived from donors I, II, and III. Enucleated cells constituted around 10–35% of the starting sample. As predicted, due to their deformable nature, the majority of enucleated cells were hydrodynamically directed to outlet A (closest to the outer wall).

The high‐quality end‐product derived from donors I and III constituted a good quality starting material for the label‐free purification resulting in highest separation efficiency (>90%) and purity (>70%). In contrast, less abundant (Figure 3a), stiffer (Figure 3b), and smaller (Figure 3c) enucleated cells derived from donor II were more troublesome to purify. Their separation efficiency and purity in outlet A were ~10% lower in comparison to donors I and III (Figure 5b). Stiffer enucleated cells from donor II, probably, experience less of the effect of F_D_, and they assemble the lateral equilibrium position closer to the channel centreline resulting in their partial capture in outlet B (separation efficiency: 12.3%, Figure 5b).

Transfusion of nucleated cells poses a leukemogenic risk (Zeuner et al., 2012), thus this product should be further purified if intended for clinical application, as a reasonable fraction of this cell type is found in outlet A. This was achieved by adding a filtration step after processing in the spiral channel (Figure 5c). Cells from donors I and II collected at the outlets A were passed through 3 µm polycarbonate Isopore™ filter membrane (see Supporting Information Mateiral; SInfo – Filter membrane characterization for justification of the filtration step) counted and assessed by flow cytometry. This enhanced the purity of enucleated cells collected at outlet A to 99%. The high purity, however, was a trade‐off for separation efficiency, since during this process, 50–70% of enucleated cells were lost. It is important to note that current processing alternatives (such as FACS/MACS) would lead to similar recoveries/purities while requiring expensive and potentially harmful immune‐labeling.

#### 3.1.4 Processing impact on cells

**Figure 6:**
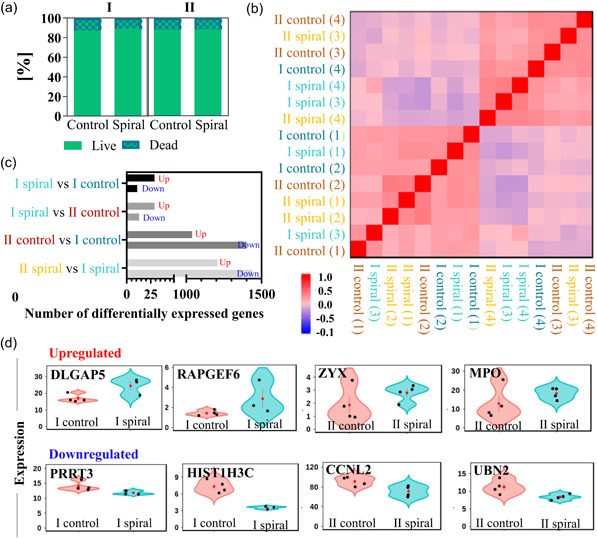
(a) Viability of mRBC derived from donor I and II, before (control) and after (spiral) processing in a spiral microchannel with 170 × 30 µm cross‐section at 1 ml/min flow rate, measured by trypan blue exclusion assays. Bars represent mean fraction of live (plain green) or dead (checkered pattern) cells found in the samples, measured at three independent experimental occasions. (b) Heatmap of sample correlation between each pairwise combination of samples, for four replicas (number of replicas is indicated in brackets). The correlations were calculated using Spearman correlation based on all gene expression values. The level of correlation (Spearman correlation coefficient) is represented by color intensity, with strong positive correlation in red, no correlation in light pink and strong anti‐correlation in blue. The plot has been hierarchically clustered on both axis using‐ there is no distinct gene expression alteration. (c) Bar chart showing the number of significantly (*p* < .05) upregulated (up) and downregulated (down) genes, between donor I (and donor II, respectively) cells processed is the spiral microchannel compared to control sample as well as donor II control and processed cells compared to control and processed cells derived from donor I. (d) Violin plots showing two most significantly (selected by the lowest *p*‐value) upregulated and downregulated genes after processing (spiral) in comparison to control cells, for both donor I and II. Each dot is one sample, with sample groups given on the x‐axis and gene expression on the y‐axis. The mean and standard error for each sample group is given as a red dot and line. The spread of the samples within a sample group gives an idea of sample heterogeneity at a given gene. mRBC, manufactured red blood cell [Color figure can be viewed at wileyonlinelibrary.com]

Inertial microfluidic techniques are considered as a gentle method for biological samples processing, with significant literature evidence supporting unaffected cell quality (e.g. viability, cell membrane integrity, proliferation, or altered gene expression) after processing (Hur et al., 2011; L. M. Lee et al., 2018; W. C. Lee et al., 2011; Nathamgari et al., 2015). However, elevated flow rates are required to benefit from the deformability‐induced lift force. To investigate if the hydrodynamically induced mechanical stress on the cells exerted any adverse effect on the mRBC, phenotype cell integrity and global gene expression profile were studied (Figure 6). The gene expression study did not include any investigation of the impact of filtering.

Populations of mRBC derived from donor I and II, at concentrations of ~3 × 10^6^ cells/ml were processed in the spiral microchannel with 170 × 30 µm^2^ cross‐section at 1 ml/min flow rate, and eluents from all outlets were collected into one vial (labeled spiral) and their quality was compared against unprocessed cells (control). Cell integrity of the control and processed cells was investigated via trypan blue exclusion assay. Live cells are impenetrable for trypan blue, while damaged cells with impaired cell membrane integrity uptake trypan blue and they appear blue. As shown in Figure 6a, the high viability of >85% was comparable at the inlet (control) and after processing (spiral).

Cells actively respond to mechanical perturbations through the modification of gene expression (Miroshnikova, Nava, & Wickstrom, 2017). To investigate if exposing undifferentiated nucleated cells to mechanical stress engages oncogenes signaling pathways, the global gene expression patterns were investigated using poly‐A selection method. Control and processed (spiral) samples were collected earlier during the differentiation process (Day 14) than samples for trypan blue assay, to ensure that nucleated cells were still transcriptionally active.

After sequencing, a standard pipeline was run that seeks to describe the variance and correlative behavior across the data before classifying those genes that are differentially expressed in each core comparison. Sample by sample correlation analysis was performed to attempt to ascertain how strongly or weakly each sample correlates across the range of gene expression values (Spearman correlation clustering, hierarchically clustered). A strong tendency for samples to cluster by sample group (donors I and II), overriding the effects of processing in the spiral microchannel (Figure 6b), was observed.

We then sought to describe the individual changes in gene expression using CuffDiff (Trapnell et al., 2013). Here, a small number of genes changing significantly (*p* < .05) as a result of cell processing—40 and 42 changing genes in samples from donors I and II, respectively, was observed. This was eclipsed by the scale of changes between sample groups with over 2,400 changing (donor I vs donor II) at both the control and treatment stages (Figure 6c).

Heatmaps of significantly differentially expressed genes (Figure S9) show that although genes change in relatively robust patterns, their expression changes are rarely recapitulated within treatment groups. In addition, as shown in the violin plots (Figure 6d), for the two most upregulated and downregulated genes (selected by the smallest *p*‐value), the level of changes are small when compared to the spread in the expression levels between replicates.

In summary, it has been confirmed that mRBC, after processing within the spiral microchannel at a sufficiently high flow rate to take advantage of the effect of F_D_ for cells focusing, retains a high degree of viability and that there is no distinct or consistent gene expression alteration.

## 4 DISCUSSION

In this study, we successfully developed a passive, high‐throughput, label‐free purification strategy for CB CD34+ derived red blood cells. Using advances in the field of deformability cytometry, heterogeneous end‐products of CB CD34+ in vitro erythropoiesis were characterized and label‐free markers were identified for the target enucleated cells as well as contaminant nucleated cells and expelled nuclei. These label‐free markers were used as a two‐step purification strategy: (a) using inertial focusing in a spiral microchannel where most of the target enucleated cells are covered (>90%) at relatively high purity (>70%), without compromising cell quality and (b) a membrane filtration step resulting in the removal of ~99% of remaining impurities (mainly nucleated cells since >98% of nuclei were removed by the spiral microchannel). The inertial focusing strategy is based upon deformability sorting. Given the size of the overlap of the enucleated and nucleated cells, the only explanation for the shift toward the outer wall of the enucleated cells is the deformability difference; this phenomena has been previously reported and characterized, and although being a novel approach, further investigation of the underlying theoretical physics, supported by experimental data, is required (Guzniczak et al., 2020). The membrane filtration step requires further optimization and development, since in this study, dead‐end filtration, which is prone to membrane fouling, led to the separation efficiency of 30–50% of enucleated cells. Shah et al. (2016) reported a positive evaluation of CB CD34+ derived mRBC as transfusion product (Shah et al., 2016), using their novel animal model to assess the potential of mRBCs to deliver oxygen to muscle tissues. To deplete undifferentiated nucleated cells before transfusion, they used a nonwoven fabric filter (Tao, Xia, Cao, & Gao, 2011). They carried out an extensive study on the impact of filtration on the quality of mRBC and they found that cells, despite a significant cell retention on the membrane (filtration removed ~75% of cells), mRBC passed through the filter remained intact and there were no difference in levels of hemoglobin expression before and after filtration. Gene expression changes were not studied by them, nor in our work.

In the demonstrated approach, >3 × 10^6^ cells/min are processed by a single device when operating at the optimal flow rate. The downstream processing method proposed in this study has the capacity for further scale‐up by two means: increasing cell sample concentration and system parallelization. However, the current cell sample concentration seems reasonable for processing cells that are routinely cultured within a similar concentration range in large volumes. At present, mRBC culture is routinely carried in static culture conditions, facilitating maximal cell concentration at around 5 × 10^6^ cells/ml (Rousseau et al., 2014). Volumetric throughput in the device presented here is 1 ml/min in a single layer system, which again is compatible with the state‐of‐the art bioreactor sizes (Rousseau et al., 2014), though larger volumes are likely to be required for commercial production. Throughput could be improved by parallelization and/or stacking, for example, like recent work by Warkiani that reached 240 ml/min or 350 L/day, with the authors reporting further parallelization was possible to triple the throughput (Warkiani, Tay, Guan, & Han, 2015). Stacking microfluidic devices (stack of 20 devices reported; Miller, Jimenez, & Bridle, 2016) is a common practice resulting in a rapid and efficient throughput improvement. Further clarification is needed on exact requirements for industrial‐scale production, though, given the lack of impact on the cells of this approach, processing time is more likely to influence the economics of the process rather than cell quality. Since the device operates at elevated follow rate to reveal the differential equilibrium position determined by deformability, one of the pragmatic challenges would be to identify a suitable pumping system, withstanding high pressures (up to 30 bars) and operating in a continuous mode. Currently, cell suspensions are introduced into the device in 10 ml batches using a mid‐pressure syringe pump.

Membrane filtration alone is less effective in processing the mRBC than the combined process consisting of processing in spiral microchannel followed by filtration. Particle separation by means of filtration is a widely applied technique within field of bioprocessing (Masri et al., 2017). Membrane filtration uses an average pore size where particles larger than the pore size cannot pass through. Traditional membrane filtration suffers from several drawbacks, with the main one being clogging. Clogged membrane filters degrade in performance over time and the “filter cake” may pose contamination hazards (Seo, Lean, & Kole, 2007). Membrane filtration is especially problematic for mRBC purification due to presence of large quantities of free‐floating nuclei. DNA is known for being “sticky” molecule and causing fouling of surfaces (Timmins & Nielsen, 2011). Inertial focusing in spiral microchannels has been proposed as “membrane‐free” filtration, capable of continuous and high‐throughput separation based on size and deformability (Bhagat et al., 2008; Guzniczak et al., 2020). By processing the sample in spiral microchannel, a majority (>98%) of the nuclei are depleted, prolonging the life‐span of the filter membrane. The dead‐end membrane filtration used in this study requires further optimization and development to improve cell recovery.

All current protocols for the manufacture of RBC from stem cells face the same technological challenge of low enucleation rate. The most efficient solution is coculture with macrophages, which eliminate the expelled nuclei by the means of phagocytosis. This is an organic solution but it comes with its own technological costs, such as finding ways to retrieve the macrophages from the culture and the complexity of a coculture system with feeder layer. In the most optimistic scenario, even if the enucleation rate is improved to reach the desirable 100% and nucleated cells are not present in the end‐product of the in vitro erythropoiesis, in the absence of macrophages, the expelled nuclei will still remain within the sample. Having a robust label‐free procedure for mRBC purification at high‐throughput with no impact on cell quality will consequently be of significant importance for bringing mRBC a step closer to clinical use.

CB CD34+ cells are a limited and variable source of mRBC and as verified in this study, starting cell material derived from different donors give a final product characterized by different phenotypes and mechanotypes, thus implementation of universal downstream protocols is currently challenging. The field of stem cell‐derived therapeutic products is maturing and with introduction of iPSC (Lapillonne et al., 2010) and immortalized erythroid cell lines (Trakarnsanga et al., 2017), it should be possible to produce large quantities of standardized mRBC and integrate technology proposed here into the formulation step of the cellular product derivation process.

To conclude, this study presents a much‐needed label‐free high‐throughput (millions of cells/min, ml of medium/min) scalable and continuous cell sorting approach for novel stem‐cell‐derived therapeutic products. In addition, the capability to sort multiple cell types simultaneously based on their size and deformability, at high‐throughputs, within one system and without the compromising effect of fluorescent labels could be highly relevant for isolation of various cells of interest from heterogeneous samples.

## CONFLICT OF INTERESTS

The authors declare that there are no conflict of interests.

## AUTHOR CONTRIBUTIONS

EG designed and performed all presented experimental work and data analysis (except gene expression profiling) and wrote the manuscript, OO contributed to the RT‐DC experiments design and writing of the manuscript, GW developed bespoke LabView software for cytospin image analysis and contributed to writing of the manuscript, TC and NR performed bioinformatic analysis and data interpretation for the gene expression profiling, NW contributed to the writing of the paper, MJ and HB equally contributed to the experimental design and writing of the article.

## Supporting information

Supplementary information
