# Supplementary material for: Purifying stem cell‐derived red blood cells: a high‐throughput label‐free downstream processing strategy based on microfluidic spiral inertial separation and membrane filtration
Source: Biotechnol Bioeng. 2020 Mar 15;117(7):2032–45. doi: 10.1002/bit.27319 (PMC7383897; doi:10.1002/bit.27319)
Supplement: Supplementary file 1 — Supplementary information [file BIT-117-2032-s001.docx]

# Supplementary Information

| Source | Culture period | Enucleation rate | Ref |
| --- | --- | --- | --- |
| **CB CD34+** | 21 days | 4% | (Neildez-Nguyen et al., 2002) |
|  | 20 days | 75-80% | (Miharada, Hiroyama, Sudo, Nagasawa, & Nakamura, 2006) |
|  | 21 days | 44-84% | (Baek et al., 2008) |
|  | - | >90% | (Nicholas E Timmins, Athanasas, Gunther, Buntine, & Nielsen, 2011) |
|  | 38 | 99.4%* | (Fujimi et al., 2008) |
| **PB CD34+** | 18 days | 68% | (Giarratana et al., 2011) |
|  | 18 days | 55-95% | (Griffiths et al., 2012) |
|  | 21 days | 63% | (Kupzig, Parsons, Curnow, Anstee, & Blair, 2017) |
| **PBMC** | 20 days | 80-90% | (Akker, Satchwell, Pellegrin, Daniels, & Toye, 2010) |
| **hESC** | 59 days | 1.5-16% | (Qiu, Olivier, Velho, & Bouhassira, 2008) |
|  | 42 days | 10-65% | (Lu et al., 2008) |
|  | 60-125 days | 2-10% | (Dias et al., 2011) |
| **hiPSC** | 26 days | 4-10% | (Lapillonne et al., 2010) |
|  | 60/125 days | 2-10% | (Dias et al., 2011) |
|  | 52 days | 20-26% | (Kobari et al., 2012) |
| **BEL-A** | 18 days | 30% | (Trakarnsanga et al., 2017) |

***STable. 1***

*Examples of published in vitro erythroid differentiation protocols and reported enucleation rates. *In 2008 Fujimi et al. reported CB CD34+ differentiation strategy providing an almost complete enucleation (99.4%)* (Fujimi et al., 2008)*, however, that was achieved by co-culture with macrophages, making the protocol unsuitable to scale-up* (Goers, Freemont, & Polizzi, 2014)*.*

***CB****- cord blood,* ***PB****- peripheral blood,* ***PBMC****- peripheral blood mononuclear cells,* ***hESC****- human embryonic stem cells, hiPSC- human induced pluripotent stem cells,* ***BEL-A****- Bristol Erythroid Line Adult*


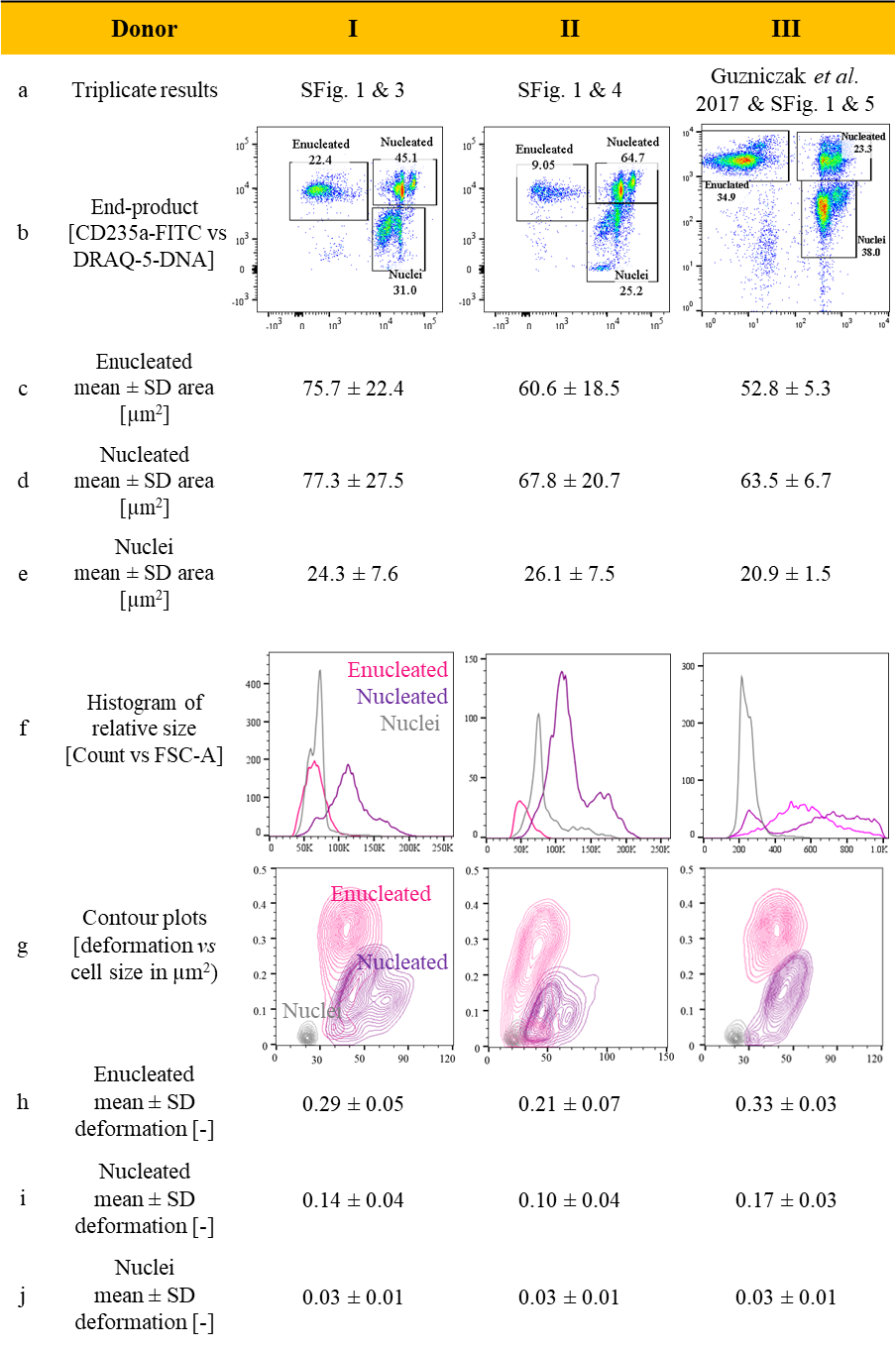


***STable 2***

*Table summarising size and deformability properties for the end product of CB CD34+ derived from three donors (donor I, donor II and donor III), in vitro erythropoiesis. (****a)*** *Donor I and II were characterised specifically for this study, donor III has been described previously in Guzniczak et al. 2017. For the clarity, scatter plots for one out of three replicas are shown- for the triplicate results please see appendices and the publication as indicated in the table.* ***(b)*** *Flow cytometric assessment of the presence of enucleated cells, nucleated cells and expelled nuclei in the end-product of the differentiation protocol. Enucleated cells are DNA- and CD235a+, nucleated cells are DNA+ and CD235a+, free-floating nuclei are DNA+ and CD235+, however, they express lower levels of CD235a than nucleated cells. Each scatter plot displays around 10000 events split accordingly between each subpopulation. To get further insight into the properties of event from each population, cytospin image analysis was performed to extract mean ± SD area of* ***(c)*** *enucleated cells* ***(d)*** *nucleated cells and* ***(e)*** *nuclei.* ***(f)*** *Histograms of FSC-A parameter reflecting relative sizes of enucleated (pink) and nucleated (purple) cells as well as the free-floating nuclei (grey), measured by flow cytometry. The number of events on each diagram is around 10,000 - split accordingly between each subpopulation.****(g)*** *Equal probability contour plots (the same number of cells fall between each pair of contour lines) of deformation vs cell size (expressed as projected cell area in µm^2^) for enucleated (pink) and nucleated (purple) cells and nuclei (grey) generated using RT-FDC (for donor I and II) and RT-DC (for donor III). From the above contour plots mean ± SD deformation of enucleated* ***(h)*** *enucleated and* ***(i)*** *nucleated cells as well as the* ***(j)*** *nuclei has been extracted.*

The enucleated and nucleated cells and the free-floating nuclei found in the end-product of the CB CD34+ differentiation protocol could be distinguished based on their size and deformability. CB CD34+, among other cell types used as a starting material, offer relatively high enucleation rate and yields providing numerous units of mRBC. However, CB CD34+ expansion potential is limited, meaning that cells obtained from one donor constitute a finite source and that creates a difficulty in adapting this approach for routine standardised processing (Esposito, 2018).

Variation in donor- and batch-dependent hematopoietic differentiation potential of CB CD34+ has not been widely addressed, hence we characterised the end product of CB CD34+ *in vitro* erythropoiesis from three donors, two described in this study (donor I- and donor II- and an additional donor III was described elsewhere (Guzniczak et al., 2017). Our findings show that there was some degree of intrinsic variability in size and deformability characteristics between them (**STable 2**- a summary table for all three donors)

In general, the size expressed as projected cell area [µm^2^] remains roughly within the same range for each donor (24.3 ± 7.6 – 77.3 ± 27.5, 26.1 ± 7.5 – 67.8 ± 20.7 and 20.9 ± 1.5 – 63.5 ± 6.7 µm^2^, for donor I, II and III, respectively). The relative size measured by flow cytometry as FSC-A parameter, show that nucleated cells always remind the largest in the samples and that was true across all three donors. It was also true across the three researched donors that there were discrepancies in the relative size of enucleated cells and nuclei. Enucleated cells derived from donor I substantially overlapped with sizes of nuclei (AUC=0.63 ± 0.6) and the little shift was towards the larger side of the spectrum. For donor II enucleated cells were the smallest within the sample, while as shown in **SFig. 1** the subpopulation of enucleated cells derived from donor III was larger than the subpopulation of the expelled nuclei (AUC=0.95) with a substantial overlap in terms of size with nucleated cells (AUC=0.56)

Despite the size differences, nuclei always remain the most rigid within the sample while the enucleated cells are the most deformable. However, the degree to which enucleated cells can deform vary between donors, mean deformation of enucleated cells derived from the best performing donor III was 0.33 ± 0.03 (Guzniczak et al., 2017), which is comparable to deformation measured for peripheral blood red cells measured in RT-DC (D=0.35 ± 0.03, **SFig. 2.** In contrast, deformation of enucleated cells derived from donor II (D=0.21 ± 0.07), which also had the lowest expansion and enucleation rate among all tested donors, was closer to the deformability of nucleated cells from donor III (D= 0.17 ± 0.03, than to enucleated cells from donor II and RBC (**SFig. 2**).


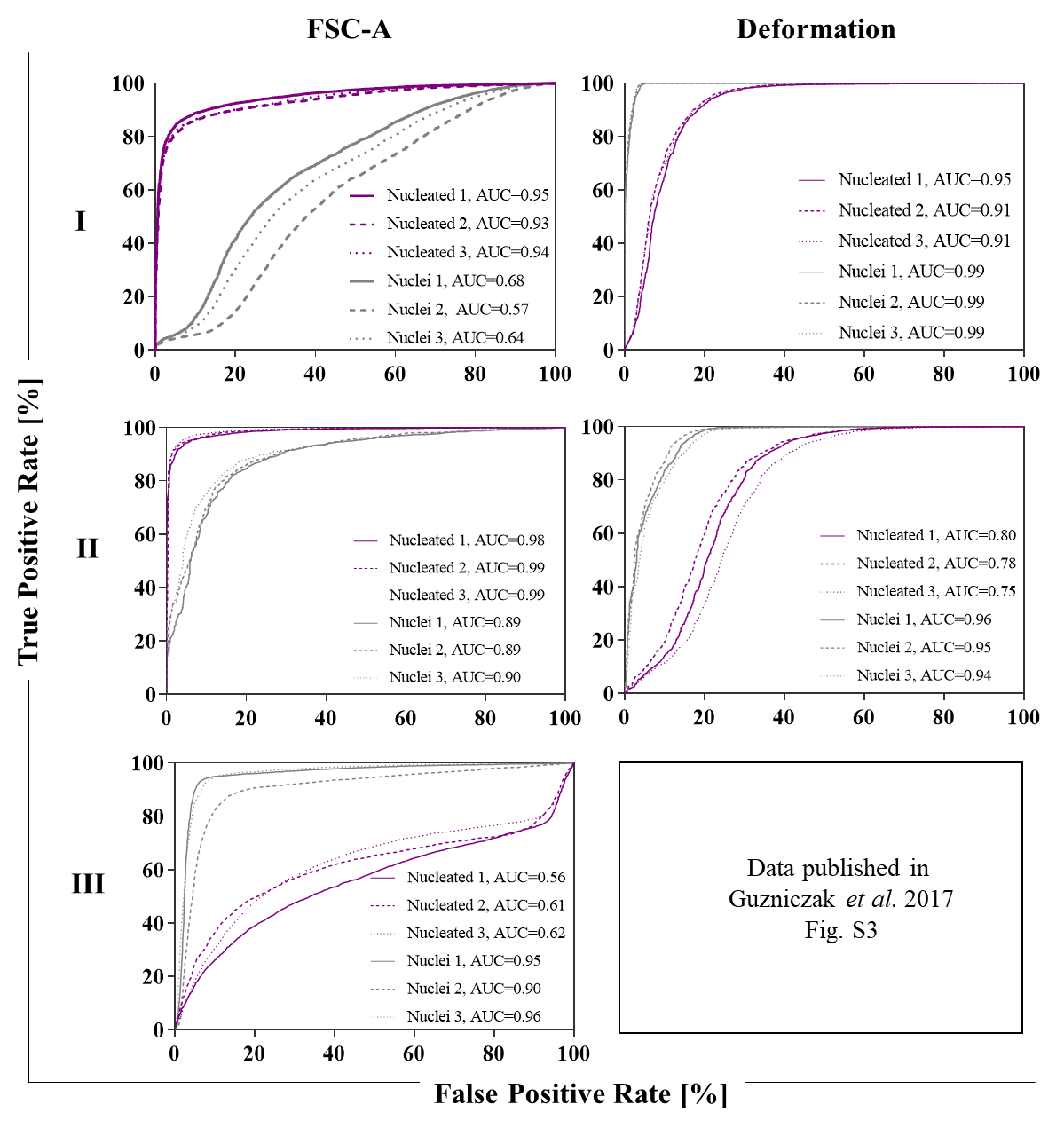


***SFig. 1***

*Receiver Operating Characteristic (ROC) curves were plotted for size (expressed as FSC-A parameter) and deformability [-] for enucleated versus nucleated cells (purple) and enucleated cells versus nuclei (grey) at the end of the differentiation protocol for three replicas (indicated as 1, 2, 3) for donor I, II and III.. The True Positive Rate is defined as the number of enucleated cells measured for a certain cut-off point (size or deformability) and divided by the total number of enucleated cells. The False Positive Rate is the corresponding number of nucleated cells (resp. nuclei) divided by the total number of enucleated cells (resp. nuclei) for the same cut-off. The Area Under the Curve (AUC) was calculated to quantify the size and deformability overlaps.*


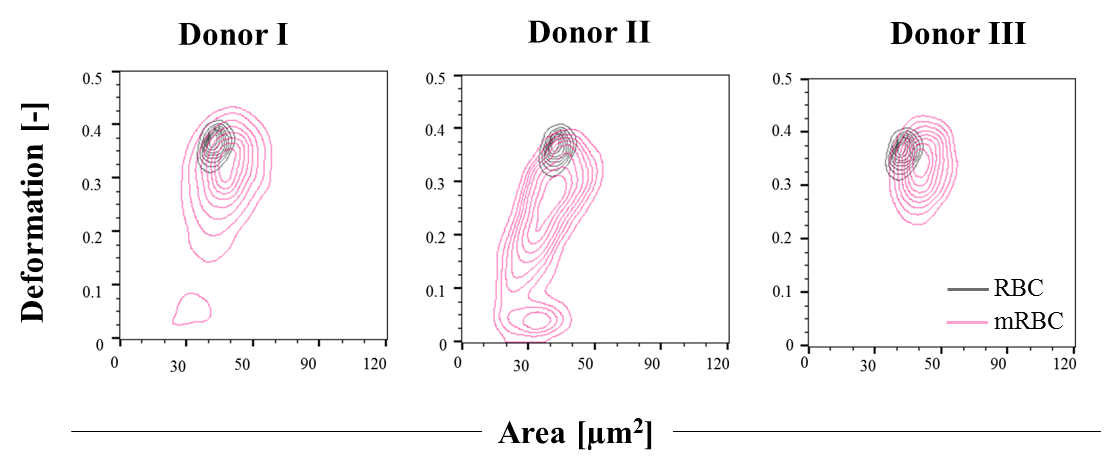


***SFig. 2***

*Comparison of mRBC derived from CB CD34+ from three donors (I, II and III) with packed RBC purchased commercially RBC from peripheral blood. Equal probability contour plots (the same number of cells fall between each pair of contour lines) of deformation vs cell size (expressed as projected cell area in µm^2^) for enucleated cells derived from each researched donor (pink) and packed RBC purchased commercially (grey).*

The differentiation process for each batch is recapitulated either exactly or with very close similarities in terms their expansion and enucleation rate, levels of expression of CD235a and well as characteristics of their label-free markers (size and deformability). However, not all researched donors had the same capacity to differentiate into enucleated mRBC. In this study, we showed that erythroid differentiation did not occur at the same levels for all studied donors.


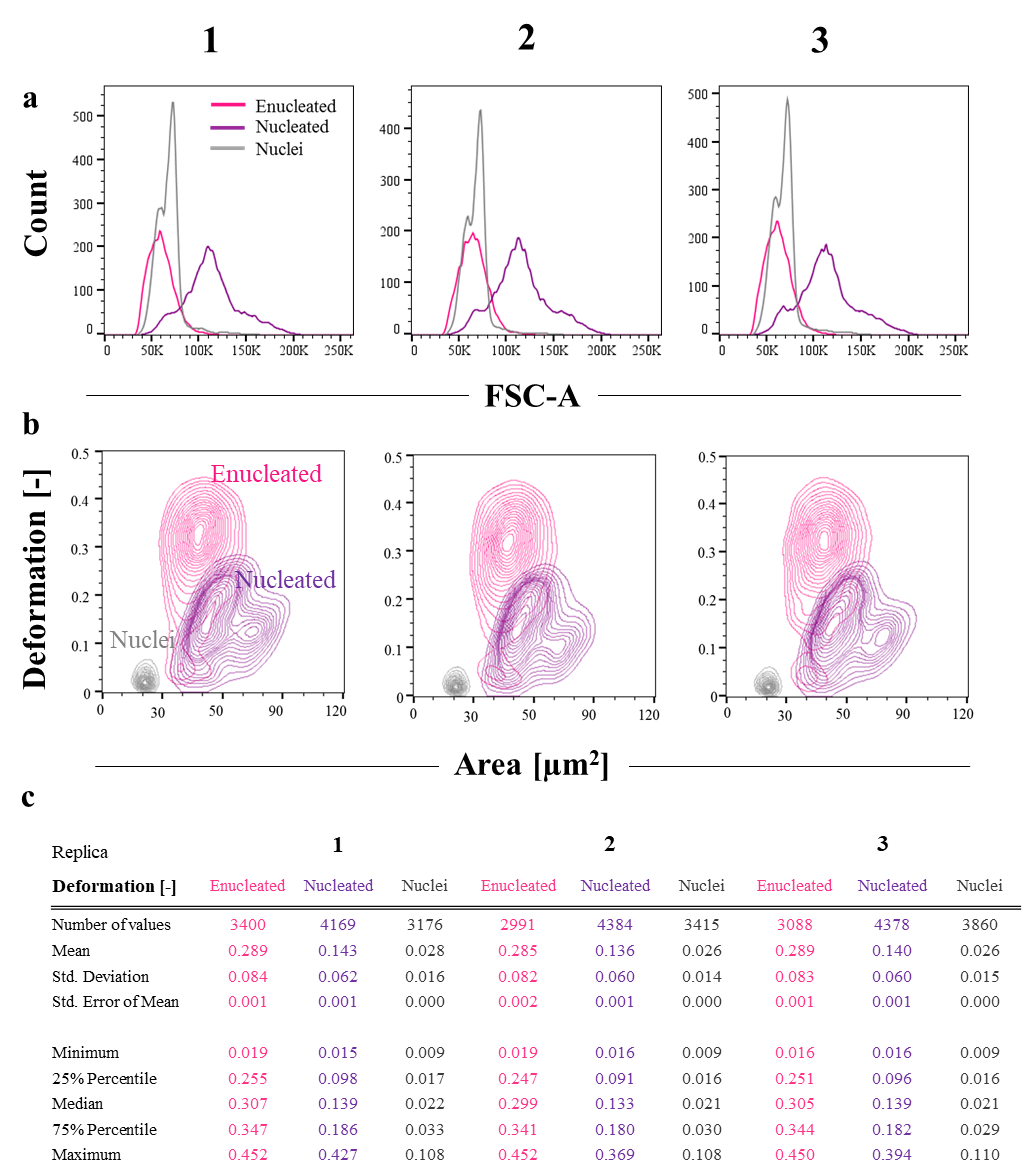


***SFig. 3***

*Three replicas (indicated as 1, 2 and 3) of flow cytometric and RT-FDC measurements of the end-product of CB CD34+ derived from* ***donor I****- in vitro differentiation into red blood cells.* ***(a)*** *Histograms of FSC-A parameter reflecting relative sizes of enucleated (pink) and nucleated (purple) cells as well as the free-floating nuclei (grey), measured by flow cytometry. The number of events on each diagram is around 10,000 - split accordingly between each subpopulation.* ***(b)*** *Equal probability contour plots (the same number of cells fall between each pair of contour lines) of deformation vs cell size (expressed as projected cell area in µm^2^) for each enucleated and nucleated cells and nuclei generated using RT-FDC for three replicas.* ***(c)*** *A summary table showing the statistical summary of deformation of each subset found in the end product of the differentiation protocol.*


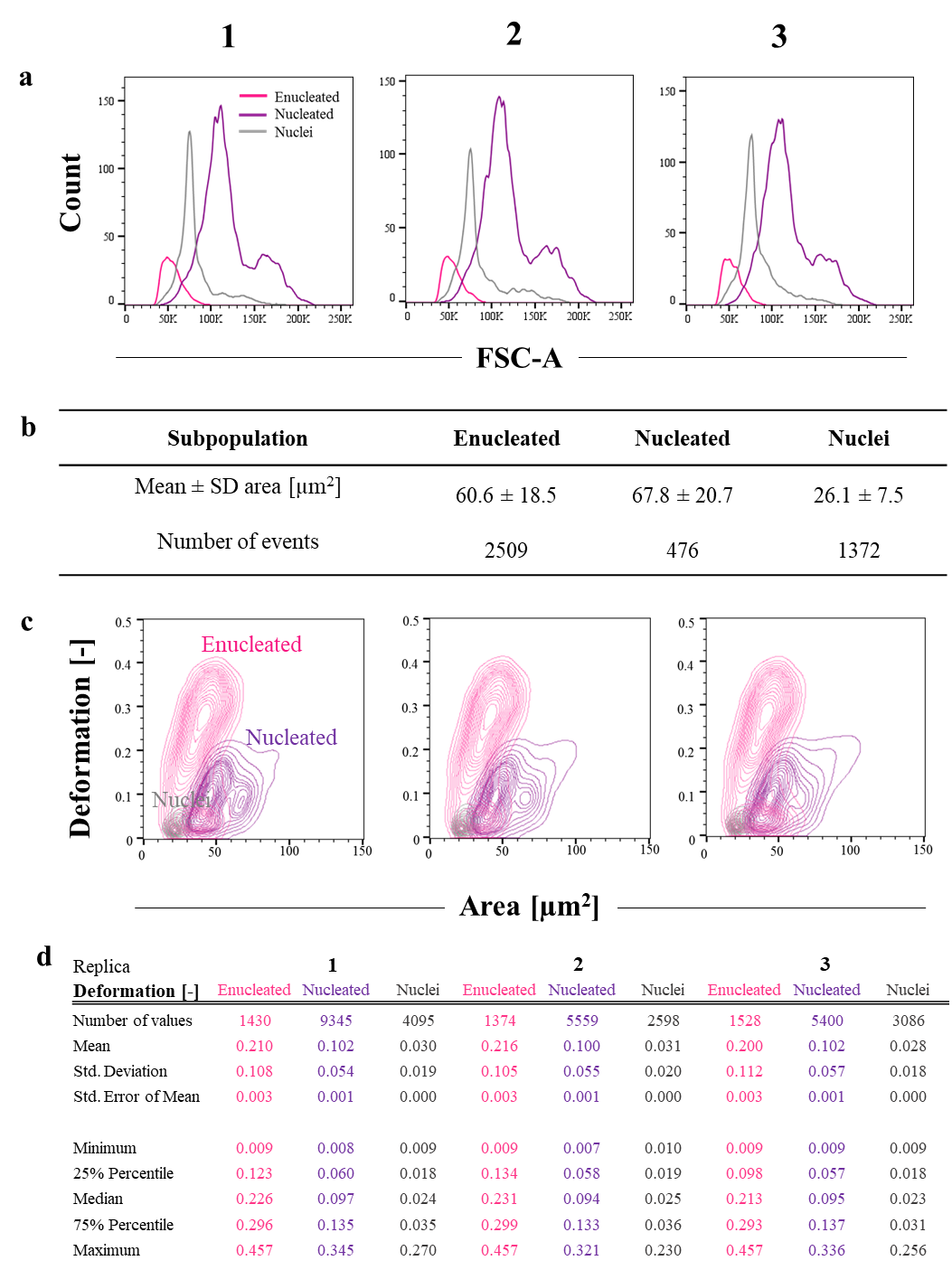


***SFig. 4***

*Three replicas (indicated as 1, 2 and 3) of flow cytometric and RT-FDC measurements of the end-product of CB CD34+ derived from* ***donor II****- in vitro differentiation into red blood cells.* ***(a)*** *Histograms of FSC-A parameter reflecting relative sizes of enucleated (pink) and nucleated (purple) cells as well as the free-floating nuclei (grey), measured by flow cytometry. The number of events on each diagram is around 10,000 - split accordingly between each subpopulation.* ***(b)*** *Equal probability contour plots (the same number of cells fall between each pair of contour lines) of deformation vs cell size (expressed as projected cell area in µm^2^) for each enucleated and nucleated cells and nuclei generated using RT-FDC for three replicas.* ***(c)*** *A summary table showing the statistical summary of deformation of each subset found in the end product of the differentiation protocol.*


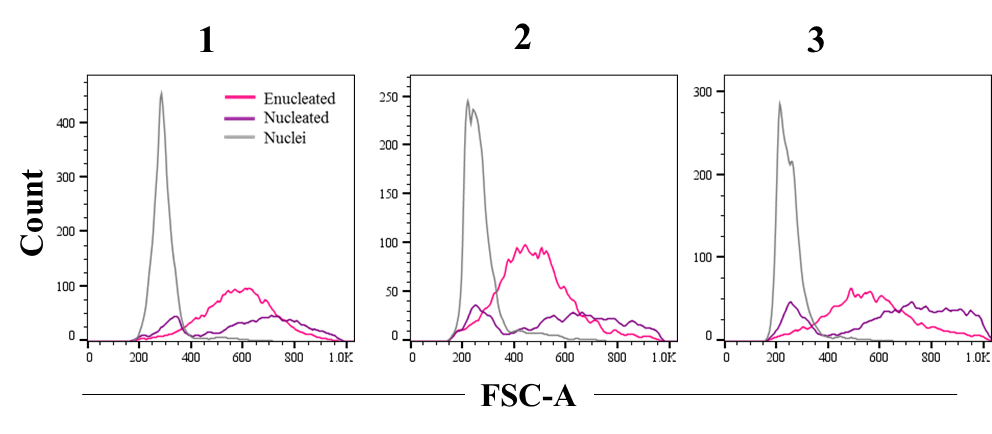


***SFig. 5***

*Relative size measurements by flow cytometry (FSC-A) for* ***donor III (a)*** *Histograms of FSC-A parameter reflecting relative sizes of enucleated (pink) and nucleated (purple) cells as well as the free-floating nuclei (grey). The number of events on each diagram is around 10000, split accordingly between each of the subpopulations.*

*
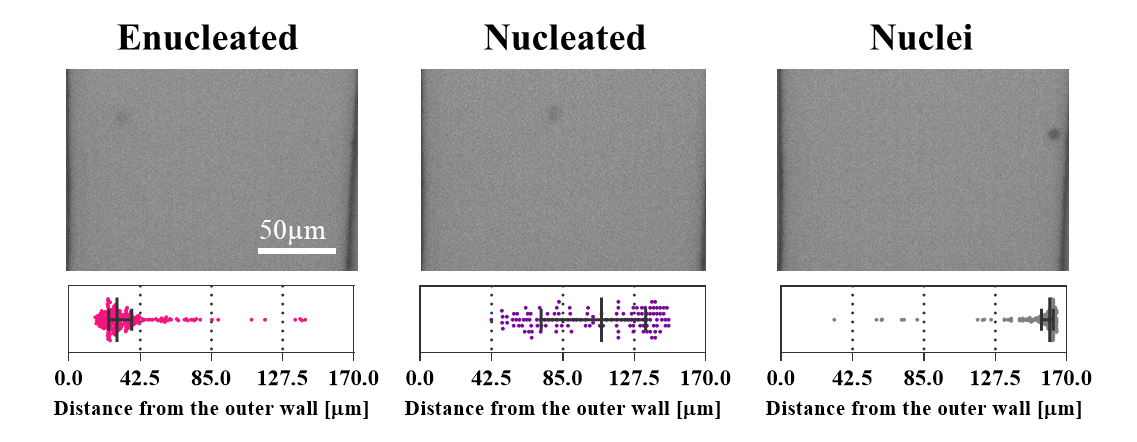
*

**SFig. 6**

*Exemplary image extracted from videos recorded for pre-sorted using FACS pure populations of enucleated and nucleated cells as well as free floating nuclei, while being processed at 1ml/min flow rate in in the spiral channel with 170 × 30 µm^2^ cross-section. Lateral equilibrium positions are measured as a distance from the outer wall (µm) at the end of the spiral channel. Data reported as median (represented as the longest vertical line) and the interquartile range (indicated by the short vertical lines) on top of scatter plots, where each dot represents one event. Around 200 events are shown for each subpopulation. Vertical dotted lines indicate four sections of the channel corresponding to four outlets of the channel (0-42.5 µm- outlet A, etc.).*

## SInfo- Filter membrane characterisation

Choosing membrane filtration as the purity enhancing step in the mRBC purification workflow was motivated with the observation that cell deformability under applied load is determined not only by cytoskeletal properties (Fletcher & Mullins, 2010) but also the presence and properties of the nucleus (Guzniczak et al., 2017)(Rosenbluth, Lam, & Fletcher, 2006). At the very last stages of the differentiation procedure, the enucleated and nucleated cells show similarities in their cytoskeleton. The actin cortex is downregulated (Hu et al., 2013) and in fluorescent microscopy, it is visible as little aggregates distributed on cell peripheries and that determines the soft nature of these cells (Guzniczak et al., 2017). The most distinct difference between enucleated and nucleated cells, however, is the presence of the nucleus. While under sufficiently high load, like during the RT-FDC experiments, the deformation of nucleated cells is limited by the presence of the nucleus (Guzniczak et al., 2017). As shown in **SFig. 7 a** when cells enter the RT-FDC measurement channel, the enucleated cells assemble the tear-like shape and their minor axis (4.3 ± 0.81, mean ± SD) perpendicular to the flow direction) is shorter than nuclei minor axis (5.0 ± 0.38), on the contrary nucleated cells assemble the bullet-like shape and their minor axis length (6.42 ± 0.9) remains above the nuclei. The nucleus is a rigid structure restricting the degree to which a nucleated cell deform from its original shape under applied load. Nuclei within nucleated cells as well as the free-floating nuclei captured in the outlet A were sized by image analysis of cytospin slides (**SFig. 7 b**). The nuclei within nucleated cells mean minor axis was 4.8 ± 0.8 µm, with the minimal measured value of 2.8 µm. The free floating nuclei were of similar sizes (minor axis 4.6 ± 07, the minimal measured value was 2.9 µm).


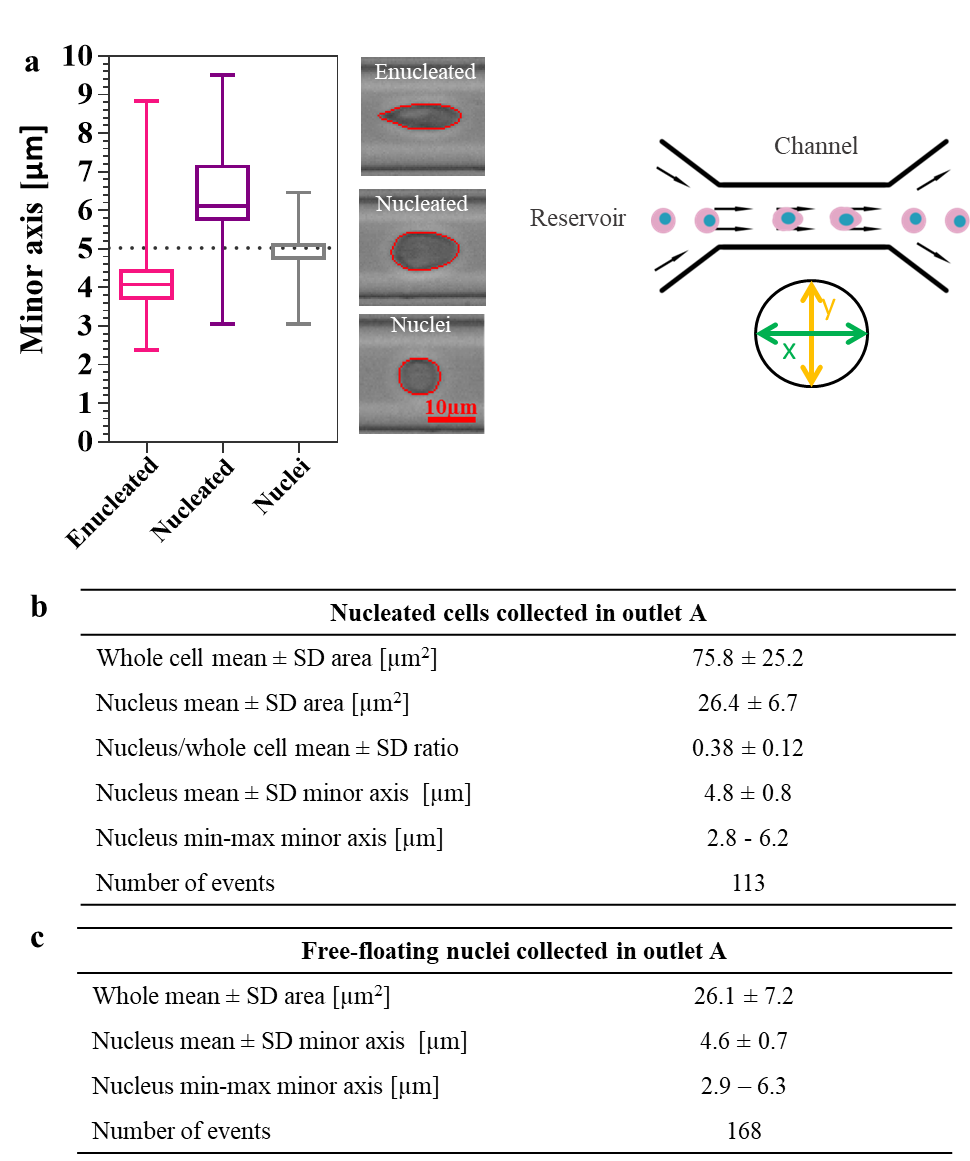


**SFig. 7**

***(a)*** *Minor axis (y-axis, perpendicular to the flow direction within the RT-DC chip) of enucleated cells (pink), nucleated cells (purple) and nuclei (grey) in the input sample, measured by RT-DC in the measurement channel section. Measurements were performed at 0.12 µl/min flow rate in a 20 µm × 20 µm^2^ channel. More than 10,000 events were acquired and split accordingly between each subset. Box plots summarising the length of minor axis [µm] for enucleated and nucleated cells and nuclei, where the line in the box represents the median, the box itself represents data from lower and upper quartile and the whiskers correspond to the lowest and highest extreme values. The dotted line indicates mean minor axis length for nuclei. To get further insight into the properties of contaminants found in the outlet A cytospin image analysis was perform to extract useful parameters describing* ***(b)*** *nucleated cell and* ***(c)*** *nuclei.*

The sizes of the free-floating nuclei and the nuclei within nucleated cells did not allow them to pass through 3 µm membrane pores and hence they should have been retained on the top of the filter.

This study did not aim to develop new membrane filtration methodology, thus initially we tested six commercially available membranes, three with 5 µm pore size and three with 3 µm pore size.

1. 5µm SVPP Durapore (Merck, cat. SVLP04700 )
2. 5µm Polyster PETE (SterliTech, cat. PET5047100)
3. 5µm NYLON (cat. NY5047100)
4. 3 µm SIGMA (Sigma, cat. 23339483)
5. 3 µm ISOPORE^TM^ (Merc, cat. TSTP04700)
6. 3 µm NUCLEPORE^TM^ (Whatman, cat. [WHA110412](https://www.sigmaaldrich.com/catalog/product/aldrich/wha110412?lang=en&region=GB))

All membranes were tested specifically with the end product of the CB CD34+ erythroid differentiation. A mixed population containing enucleated and nucleated cell as well as nuclei was passed through the filter membranes fitted onto syringe adapter and cell material retention on the membranes was assessed by performing a cell count (**STable 3**). 5µm NYLON and 3 µm SIGMA were discriminated from further tests due to high retention, 99.95% and 99.75%, respectively, of input cells, did not go through the membrane. The cut-off point of the 5 µm Polyster PETE membrane was larger than the largest cells found in the input sample and that resulted in 100% of all components of the input sample passing through the filter.


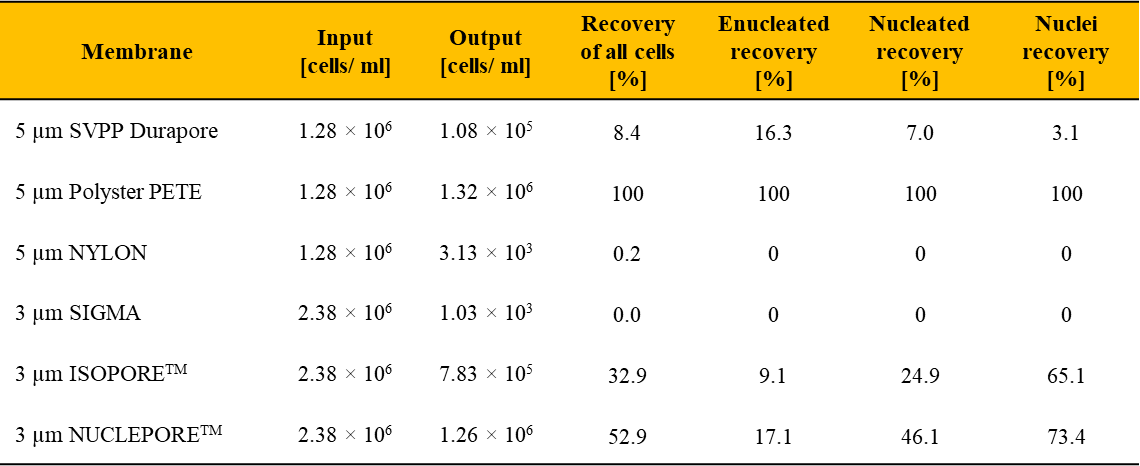


***STable 3***

*Summary of performance of the six initially tested filter membrane. Membrane type column indicates pore size and brand name of tested membranes. Input and output columns show cell concentration before and after filtration, respectively, and recovery column indicates a fraction of input sample that passed through the filter with no distinction into the three subsets.*

Input sample, as well as samples collected after filtration through the best performing 3 µm ISOPORE^TM^, 3 µm NUCLEPORE^TM^ and 5µm SVPP Durapore membranes, were characterised using standard flow cytometric assay (**SFig. 8**). In order to identify the most suitable membrane, sizes of enucleated and nucleated cells, as well as nuclei from samples collected after filtration, were compared with sizes of their counterpart subsets found in the outlet A, after processing in the spiral channel. Box plots of FSC-A parameter were generated separately for enucleated (**SFig. 8 a**) and nucleated (**SFig. 8 b**) cells and the free-floating nuclei (**SFig. 8 c**), then ROC cures were generated and AUC was calculated (**SFig. 8 d, e & f**).

The assumption for desired enucleated cells was that AUC for the best performing membrane should be either closest to 0.5 (meaning exactly the same size range). In contrast, for contaminant particles, like nucleated cells and nuclei, AUC should approach 1. Based on these assumptions we found that the best performing membrane was the 3 µm ISOPORE^TM^, the size range of enucleated cells passing through the membrane was in good agreement with enucleated cells found in the outlet A (AUC=0.54, **SFig. 8 a**), which should minimise the additional cell losses.

**
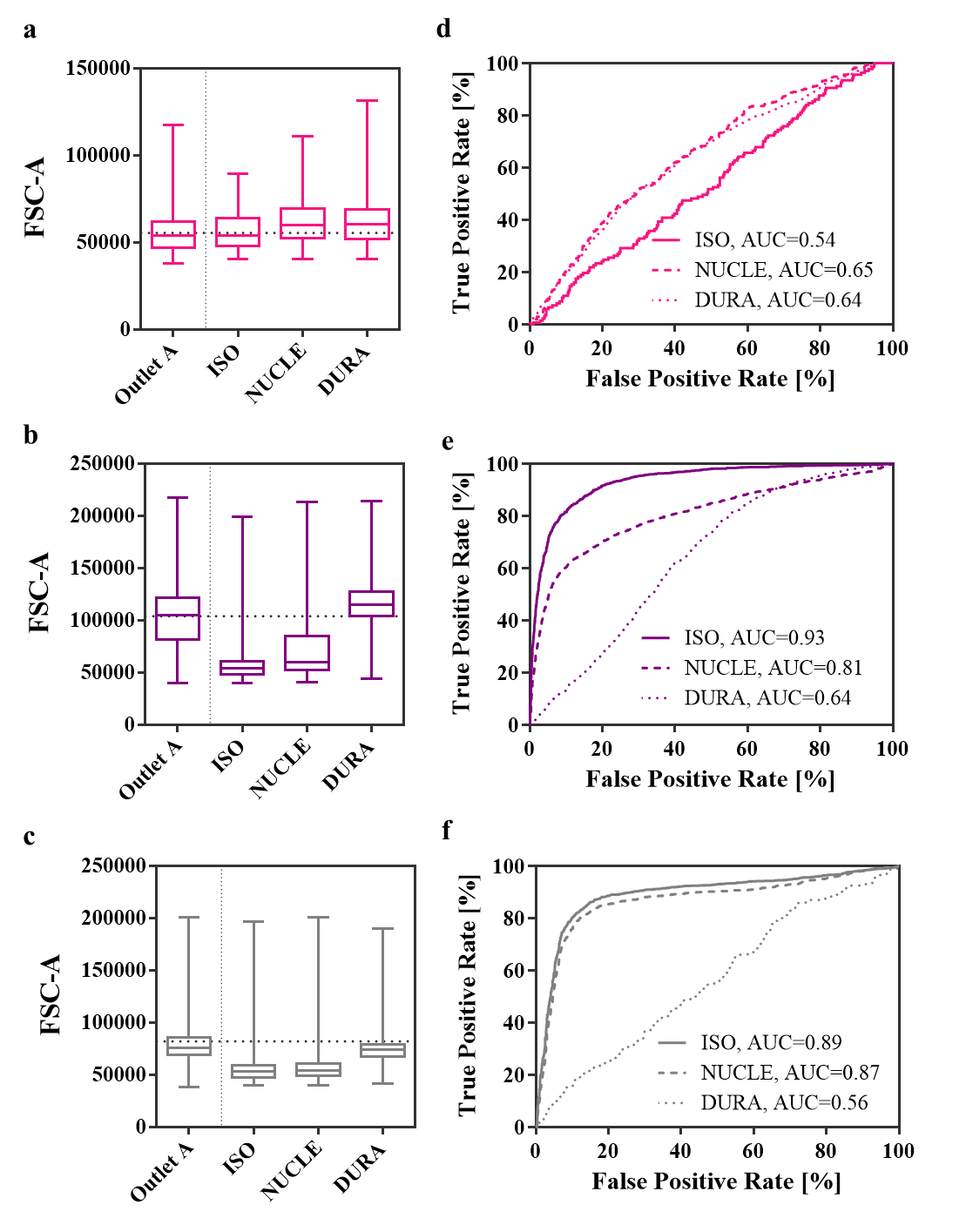
**

***SFig.8***

*Comparison of sizes of* ***(a)*** *enucleated cells,* ***(b)*** *nucleated cells and* ***(c)*** *free floating nuclei captured in the outlet A and after passing through 3µm ISOPORE^TM^ (ISO), 3µm NUCLEPORE^TM^ (NUCLE) and 5µm SVPP Durapore (DURA). Box plots summarising the FSC-A parameter indicating relative sizes for enucleated and nucleated cells and nuclei, where the line in the box represents the median, the box itself represents data from lower and upper quartile and the whiskers correspond to the lowest and highest extreme values. The dotted line indicates mean FSC-A for an adequate subset found in the outlet A. Receiver Operating Characteristic (ROC) curves were plotted for the sizes expressed as FSC-A parameters comparison between* ***(d)*** *enucleated cells,* ***(e)*** *nucleated cells and* ***(f)*** *nuclei found in the outlet A and passed through passing through 3µm ISOPORE^TM^ (solid line), 3µm NUCLEPORE^TM^ (dashed line) and 5µm SVPP Durapore (dotted line). The True Positive Rate is defined as the number of events (enucleated/ nucleated/ nuclei) found in the outlet A for a certain FSC-A cut-off point divided by the total number of events (enucleated/ nucleated/ nuclei, respectively). The False Positive Rate is defined as the number of events (enucleated/ nucleated/ nuclei) found in samples after filtration, divided by the total number of events (enucleated/ nucleated/ nuclei, respectively). In order to quantify size overlap, the Area Under the Curve (AUC) was calculated.*


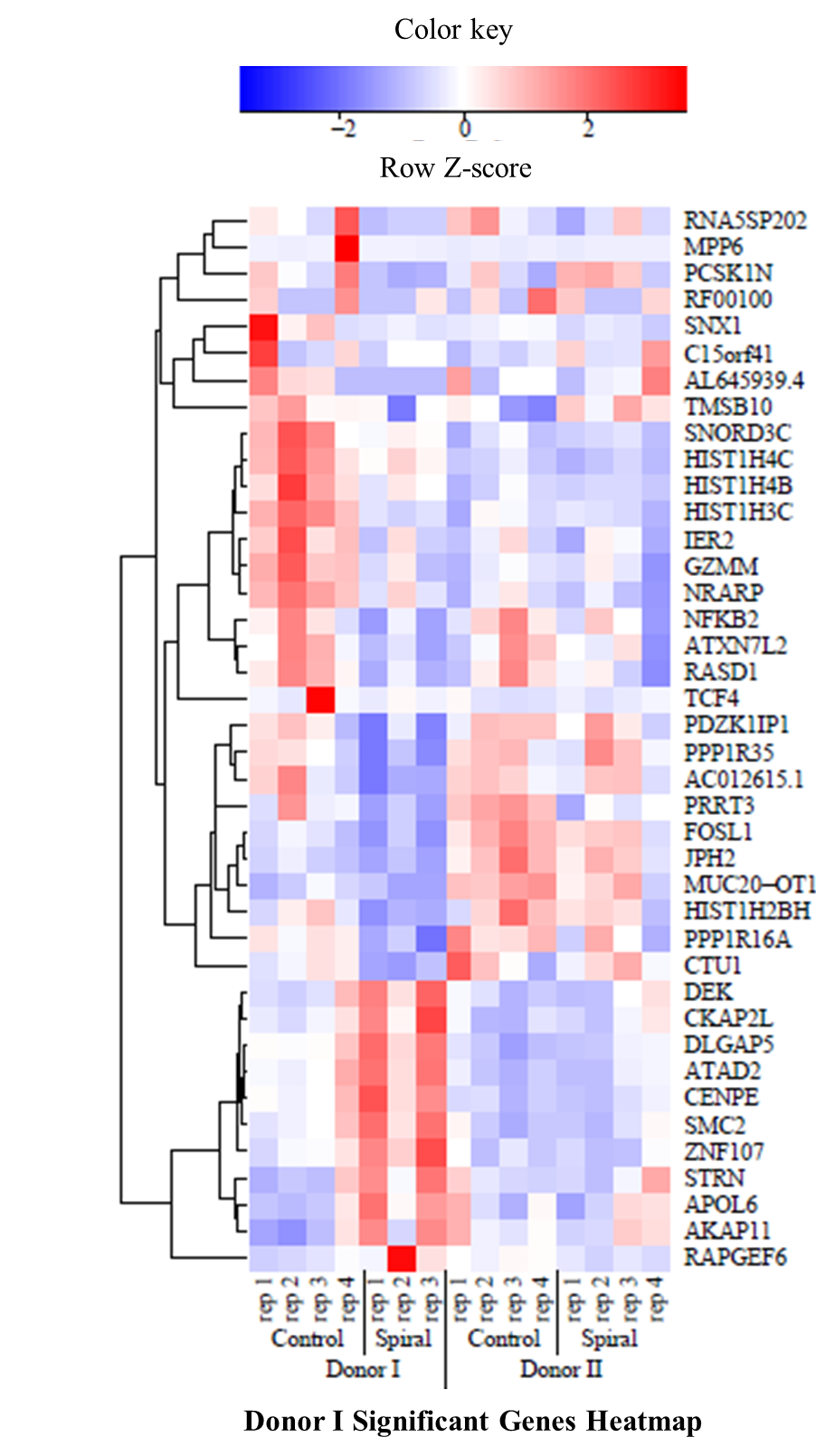


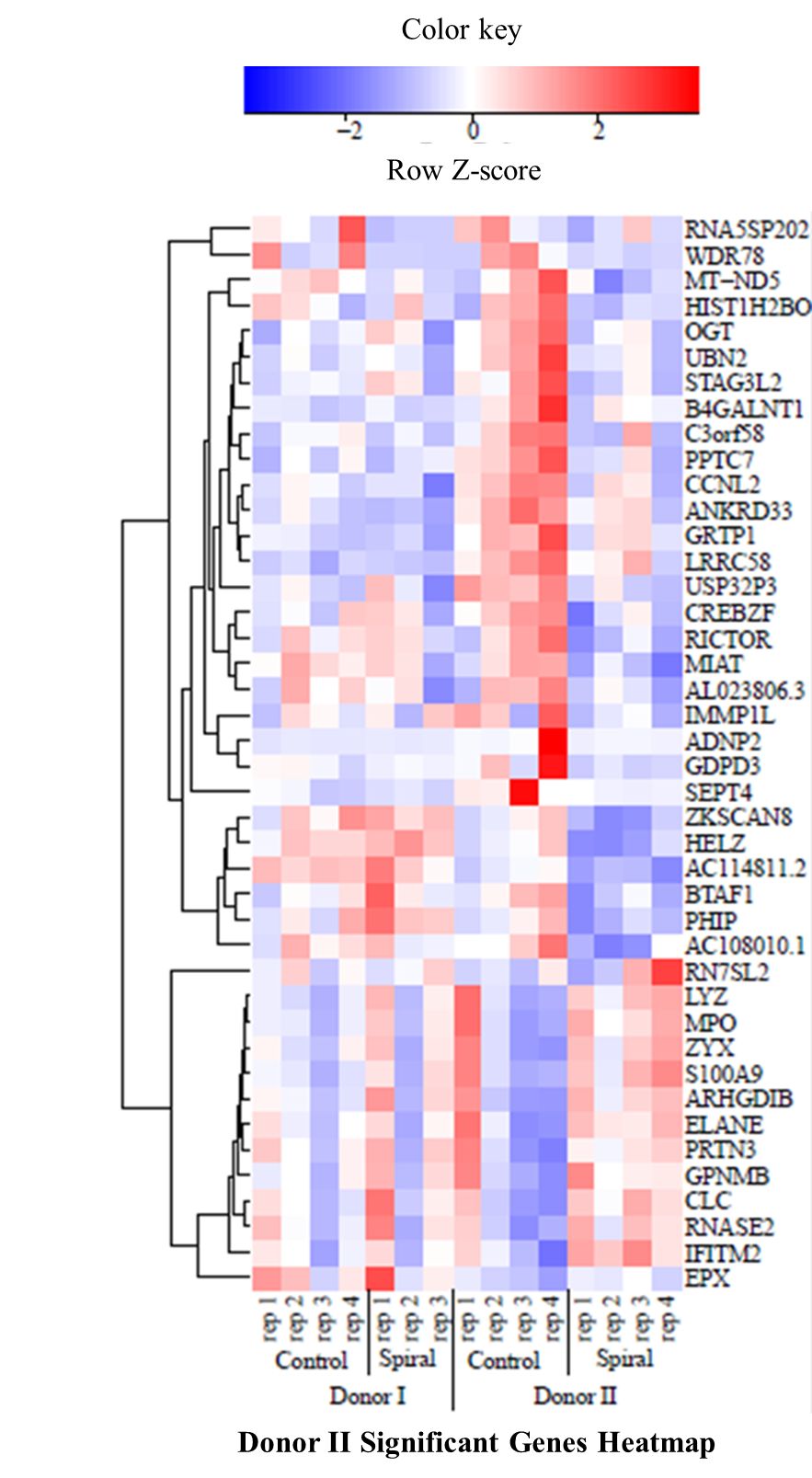


***SFig. 9***

*Hierarchically clustered heatmap of the significantly differentially expressed genes for the comparisons: donor I spiral and donor I control and donor II spiral and donor II control, for all sample in the sample groups: donor I spiral, donor I control, donor II spiral and donor II control. Samples are on the x axis and genes on the y axis. Colour intensity represents expression level, with blue representing low expression, and red representing high expression. Expression levels have been row scaled into z−scores. The y-axis (both plots) and x-axis (right plot) have been hierarchically clustered using, Spearman distances, with UPMGA agglomeration and mean reordering.*


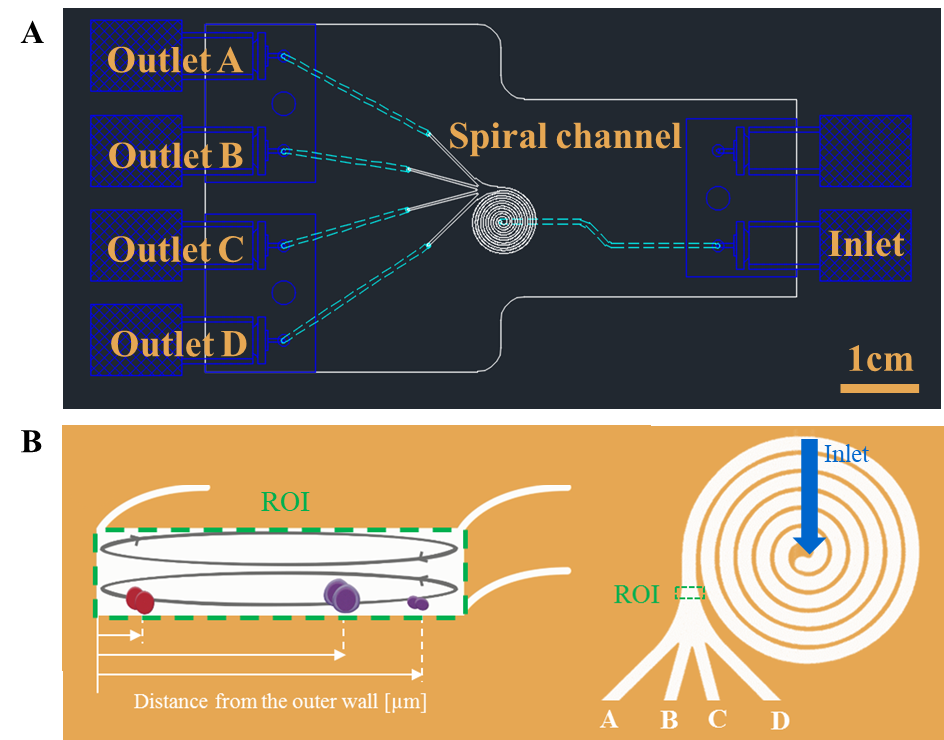


***SFig. 10***

***(A)*** *Schematic of the spiral channel with six loops, one inlet and four outlets for size and deformability-based separation. Scale bar corresponds to 1 cm.****(B)*** *Cells are introduced into the system via inlet. Hydrodynamic behaviour of cells is assessed in the end of the spiral channel in the ROI and it is expressed as lateral position within the cross-section of the channel, measured as a distance from the outer wall of the channel [µm].*

**Supplementary materials and methods**

**Real-time fluorescence and deformability cytometry**

While there are many available well-established technologies for assessing cell mechanotype such as Atomic Force Microscopy (AFM) (Vahabi, Nazemi Salman, & Javanmard, 2013), micropipette aspiration (Darling et al., 2009), magnetic tweezers and optical stretchers (Guck et al., 2001), these methods suffer from low-throughput (Musielak, 2009). To assess a high number of cells (thousands of events per minute), we used a microfluidic-based Real-Time Deformability Cytometer (RT-DC) (Otto et al., 2015). RT-DC is a contactless technique, allowing gain of thousands of events per minute, which is convenient for the global characterisation of complex samples (Xavier et al., 2016). In the RT-DC set-up, a PDMS (Polydimethylsiloxane) channel consisting of three sections, two reservoir sections and one constriction channel (20 µm × 20 µm or 30 µm × 30 µm cross section), where cells undergo deformation and measurements are undertaken. The microfluidic chip is mounted on a microscope. A syringe pump is used to pump cells suspension in the chip, pulsing LED light enables high-speed image acquisition (4000 fps), for a standard measurements, the images are acquired at ×40 magnification. Cells are introduced in the chip through central reservoir channel and they are directed into the measurement channel by sheath flow (both flow liquid and cell carrier are viscous solution of methylcellulose). Measurement channel has a cross-section slightly bigger than the cell diameter, thus cells entering the channel experiences shear stress that causes cell deformation. The images are captured in the Region of Interest (ROI) at the end of the measurement channel and processed in real time.

The RT-DC system employs image processing algorithms which enable the measurement of cell area and deformation. Deformation$(D$) is expressed as a deviation from a perfect circle

|  | $D=1-c$ | (5) |
| --- | --- | --- |

where $c$ is the circularity defined as

|  | $c=2\sqrt{\pi A}/l$ | (6) |
| --- | --- | --- |

$A$ being the projected cell area and $l$ the cell perimeter

Real-time fluorescence and deformability cytometry (RT-FDC) is an enhanced high-throughput (thousands of events per minute) microfluidic platform that enables mechanotype analysis of cells within a heterogeneous sample with no necessity of pre-sorting into pure populations, due to the integration of fluorescent signal for confirmation of cell identity (Rosendahl et al., 2018). As in the conventional real-time deformability cytometry (RT-DC) (Otto et al., 2015), cells are deformed in a contactless manner by experiencing shear stress generated by flowing in a viscous buffer through the measurement channel which is only slightly larger than the actual cell dimensions. In RT-FDC **(1)** Immuno-labelled cells are introduced into the microfluidic chip mounted on a microscope and while passing through the measurement channel **(2)** in the ROI they are imaged by bright-field microscopy **(3)**. Information about cells size (expressed as projected cell area [µm^2^]) and induced by applied shear stress deformability (understood as 1- circularity) is generated by image processing in real time for each captured event and reported as a scatter plot. Additionally, cells passing through the ROI are illuminated by focused lasers **(4)** which excite signal detected and measured in the detector array. **(5)** The fluorescent signal is correlated with the acquired image, which allows cell identity confirmation.

# Supplementary References

Akker, E. Van Den, Satchwell, T. J., Pellegrin, S., Daniels, G., & Toye, A. M. (2010). The majority of the in vitro erythroid expansion potential resides in CD34 – cells , outweighing the contribution of CD34 + cells and significantly increasing the erythroblast yield from peripheral blood samples. *Haematologica*, *95*(9), 1594–1598. https://doi.org/10.3324/haematol.2009.019828

Baek, E. J., Kim, H., Kim, S., Jin, H., Choi, T., & Kim, H. O. (2008). In vitro clinical‐grade generation of red blood cells from human umbilical cord blood CD34+ cells. *Transfusion*, *48*(October), 2235–2245. https://doi.org/10.1111/j.1537-2995.2008.01828.x

Bhagat, A. A. S., Kuntaegowdanahalli, S. S., & Papautsky, I. (2008). Continuous particle separation in spiral microchannels using Dean flows and differential migration. *Lab on a Chip*, *8*(11), 1906–1914. https://doi.org/10.1039/b807107a

Darling, E. M., Pritchett, P. E., Evans, B. A., Superfine, R., Zauscher, S., & Guilak, F. (2009). Mechanical properties and gene expression of chondrocytes on micropatterned substrates following dedifferentiation in monolayer. *Cellular and Molecular Bioengineering*, *2*(3), 395–404.

Dias, J., Gumenyuk, M., Kang, H., Vodyanik, M., Yu, J., Thomson, J. A., & Slukvin, I. I. (2011). Generation of red blood cells from human induced pluripotent stem cells. *Stem Cells and Development*, *20*(9), 1639–1647. https://doi.org/10.1089/scd.2011.0078

Esposito, M. T. (2018). Blood factory: which stem cells? *BMC Hematology*, *18*, 10. https://doi.org/10.1186/s12878-018-0105-4

Fletcher, D. A., & Mullins, R. D. (2010). Cell mechanics and the cytoskeleton. *Nature*, *463*, 485.

Fujimi, A., Matsunaga, T., Kobune, M., Kawano, Y., Nagaya, T., Tanaka, I., … Niitsu, Y. (2008). Ex vivo large-scale generation of human red blood cells from cord blood CD34+ cells by co-culturing with macrophages. *International Journal of Hematology*, *87*(4), 339–350. https://doi.org/10.1007/s12185-008-0062-y

Giarratana, M.-C., Rouard, H., Dumont, A., Kiger, L., Safeukui, I., Le Pennec, P.-Y., … Douay, L. (2011). Proof of principle for transfusion of in vitro–generated red blood cells. *Blood*, *118*(19), 5071–5079. https://doi.org/10.1182/blood-2011-06-362038

Goers, L., Freemont, P., & Polizzi, K. M. (2014). Co-culture systems and technologies: taking synthetic biology to the next level. *Journal of the Royal Society, Interface*, *11*(96), 20140065. https://doi.org/10.1098/rsif.2014.0065

Golfier, S., Rosendahl, P., Mietke, A., Herbig, M., Guck, J., & Otto, O. (2017). High-throughput cell mechanical phenotyping for label-free titration assays of cytoskeletal modifications. *Cytoskeleton*, *74*(8), 283–296. https://doi.org/10.1002/cm.21369

Griffiths, R. E., Kupzig, S., Cogan, N., Mankelow, T. J., Betin, V. M. S., Trakarnsanga, K., … Anstee, D. J. (2012). Maturing reticulocytes internalize plasma membrane in glycophorin A-containing vesicles that fuse with autophagosomes before exocytosis. *Blood*, *119*(26), 6296–6306. https://doi.org/10.1182/blood-2011-09-376475

Guck, J., Ananthakrishnan, R., Mahmood, H., Moon, T. J., Cunningham, C. C., & Kas, J. (2001). The optical stretcher: a novel laser tool to micromanipulate cells. *Biophysical Journal*, *81*(2), 767–784. https://doi.org/10.1016/S0006-3495(01)75740-2

Guzniczak, E., Mohammad Zadeh, M., Dempsey, F., Jimenez, M., Bock, H., Whyte, G., … Bridle, H. (2017). High-throughput assessment of mechanical properties of stem cell derived red blood cells, toward cellular downstream processing. *Scientific Reports*, *7*(1), 14457. https://doi.org/10.1038/s41598-017-14958-w

Herbig, M., Mietke, A., Müller, P., & Otto, O. (2018). Statistics for real-time deformability cytometry : clustering , dimensionality reduction and significance testing. *Biomicrofluidics*, *042214*(12), 1–37. https://doi.org/10.1063/1.5027197

Hu, J., Liu, J., Xue, F., Halverson, G., Reid, M., Guo, A., … An, X. (2013). Isolation and functional characterization of human erythroblasts at distinct stages: implications for understanding of normal and disordered erythropoiesis in vivo. *Blood*, *121*(16), 3246–3253. https://doi.org/10.1182/blood-2013-01-476390

Kobari, L., Yates, F., Oudrhiri, N., Francina, A., Kiger, L., Mazurier, C., … Douay, L. (2012). Human induced pluripotent stem cells can reach complete terminal maturation: in vivo and in vitro evidence in the erythropoietic differentiation model. *Haematologica*, *97*(12), 1795–1803. https://doi.org/10.3324/haematol.2011.055566

Kupzig, S., Parsons, S. F., Curnow, E., Anstee, D. J., & Blair, A. (2017). Superior survival of ex vivo cultured human reticulocytes following transfusion into mice. *Haematologica*, *102*(3), 476–483. https://doi.org/10.3324/haematol.2016.154443

Lapillonne, H., Kobari, L., Mazurier, C., Tropel, P., Giarratana, M.-C., Zanella-Cleon, I., … Douay, L. (2010). Red blood cell generation from human induced pluripotent stem cells: perspectives for transfusion medicine. *Haematologica*, *95*(10), 1651–1659. https://doi.org/10.3324/haematol.2010.023556

Lu, S.-J., Feng, Q., Park, J. S., Vida, L., Lee, B.-S., Strausbauch, M., … Lanza, R. (2008). Biologic properties and enucleation of red blood cells from human embryonic stem cells. *Blood*, *112*(12), 4475–4484. https://doi.org/10.1182/blood-2008-05-157198

Miharada, K., Hiroyama, T., Sudo, K., Nagasawa, T., & Nakamura, Y. (2006). Efficient enucleation of erythroblasts differentiated in vitro from hematopoietic stem and progenitor cells. *Nature Biotechnology*, *24*(10), 1255–1257. https://doi.org/10.1038/nbt1245

Miller, B., Jimenez, M., & Bridle, H. (2016). *Cascading and Parallelising Curvilinear Inertial Focusing Systems for High Volume, Wide Size Distribution, Separation and Concentration of Particles*. *6*, 36386. Retrieved from http://dx.doi.org/10.1038/srep36386

Musielak, M. (2009). Red blood cell-deformability measurement: review of techniques. *Clinical Hemorheology and Microcirculation*, *42*(1), 47–64. https://doi.org/10.3233/CH-2009-1187

Neildez-Nguyen, T. M. A., Wajcman, H., Marden, M. C., Bensidhoum, M., Moncollin, V., Giarratana, M.-C., … Douay, L. (2002). Human erythroid cells produced ex vivo at large scale differentiate into red blood cells in vivo. *Nature Biotechnology*, *20*(5), 467–472. https://doi.org/10.1038/nbt0502-467

Otto, O., Rosendahl, P., Mietke, A., Golfier, S., Herold, C., Klaue, D., … Guck, J. (2015). Real-time deformability cytometry: on-the-fly cell mechanical phenotyping. *Nat Meth*, *12*(3), 199–202. Retrieved from http://dx.doi.org/10.1038/nmeth.3281

Qiu, C., Olivier, E. N., Velho, M., & Bouhassira, E. E. (2008). Globin switches in yolk sac-like primitive and fetal-like definitive red blood cells produced from human embryonic stem cells. *Blood*, *111*(4), 2400–2408. https://doi.org/10.1182/blood-2007-07-102087

Rosenbluth, M. J., Lam, W. A., & Fletcher, D. A. (2006). Force microscopy of nonadherent cells: a comparison of leukemia cell deformability. *Biophysical Journal*, *90*(8), 2994–3003. https://doi.org/10.1529/biophysj.105.067496

Rosendahl, P., Plak, K., Jacobi, A., Kraeter, M., Toepfner, N., Otto, O., … Guck, J. (2018). Real-time fluorescence and deformability cytometry. *Nature Methods*, *15*(5), 355–358. https://doi.org/10.1038/nmeth.4639

Seo, J., Lean, M. H., & Kole, A. (2007). Membrane-free microfiltration by asymmetric inertial migration. *Applied Physics Letters*, *91*(3), 33901. https://doi.org/10.1063/1.2756272

Timmins, N E, & Nielsen, L. K. (2011). Manufactured RBC--rivers of blood, or an oasis in the desert? *Biotechnology Advances*, *29*(6), 661–666. https://doi.org/10.1016/j.biotechadv.2011.05.002

Timmins, Nicholas E, Athanasas, S., Gunther, M., Buntine, P., & Nielsen, L. K. (2011). Ultra-high-yield manufacture of red blood cells from hematopoietic stem cells. *Tissue Engineering. Part C, Methods*, *17*(11), 1131–1137. https://doi.org/10.1089/ten.TEC.2011.0207

Trakarnsanga, K., Griffiths, R. E., Wilson, M. C., Blair, A., Satchwell, T. J., Meinders, M., … Frayne, J. (2017). An immortalized adult human erythroid line facilitates sustainable and scalable generation of functional red cells. *Nature Communications*, *8*, 14750. Retrieved from http://dx.doi.org/10.1038/ncomms14750

Vahabi, S., Nazemi Salman, B., & Javanmard, A. (2013). Atomic force microscopy application in biological research: a review study. *Iranian Journal of Medical Sciences*, *38*(2), 76–83.

Whyte Graeme, Willoughby Nik, Jimenez Melanie, B. H. (2019). Deformability-induced lift force in spiral microchannels for cell separation. *Lab on a Chip*, *Accepeted*.

Xavier, M., Rosendahl, P., Herbig, M., Krater, M., Spencer, D., Bornhauser, M., … Otto, O. (2016). Mechanical phenotyping of primary human skeletal stem cells in heterogeneous populations by real-time deformability cytometry. *Integrative Biology : Quantitative Biosciences from Nano to Macro*, *8*(5), 616–623. https://doi.org/10.1039/c5ib00304k
